# Hairy Roots as Producers of Coumarins, Lignans, and Xanthones

**DOI:** 10.3390/molecules30173596

**Published:** 2025-09-03

**Authors:** Janusz Malarz, Iga Ryngwelska, Anna Stojakowska

**Affiliations:** Maj Institute of Pharmacology, Polish Academy of Sciences, Smętna Street 12, 31-343 Kraków, Poland; malarzj@if-pan.krakow.pl (J.M.); ryngwel@if-pan.krakow.pl (I.R.)

**Keywords:** *Agrobacterium rhizogenes*, *Cichorium*, *Gentiana*, *Hypericum*, *Isatis*, *Linum*, 6-methoxypodophyllotoxin, *Rhizobium rhizogenes*, *Sesamum*

## Abstract

Despite the great structural diversity, plant lignans, coumarins, and xanthones share numerous biological activities, ranging from antimicrobial, anti-inflammatory, and antioxidant to antineoplastic and neuroprotective. The compounds, products of the shikimic acid biosynthetic pathway, also play an important role in plant–environment interactions. In a search for sustainable and renewable sources of these valuable plant products, numerous in vitro culture systems were investigated, including hairy root cultures. The *Rhizobium rhizogenes*-transformed root cultures of over 40 plant species representing 17 families of the plant kingdom were studied in this respect. The present review focuses on the hairy roots that may be efficient producers of valuable plant products with the prospect of use in the pharmaceutical, food, or cosmetics industry. In vitro culture systems based on hairy roots, which were used to elucidate the biosynthesis pathways of the high-added-value plant compounds, were also considered.

## 1. Introduction

Hairy root (HR) cultures, since the 1980s, have become a popular tool for studies on plant metabolism [1]. The genetically stable axenic root cultures, obtained by inoculation of a plant material with either wild or genetically engineered strains of *Rhizobium rhizogenes* (also known as *Agrobacterium rhizogenes*) [2] turned out to be good producers of both biomass and specialized plant metabolites. In nature, *R. rhizogenes*, a Gram-negative bacterium, is the etiological agent causing “hairy root disease”. The mechanism of HR initiation relies on a transfer and the permanent incorporation of a foreign genetic material, delivered by the fragment (T-DNA) of the bacterial Ri plasmid, into the plant genomic DNA, which leads to a change in the hormonal balance resulting in the appearance of roots with a specific growth, structure, and metabolism. HR cultures may be used for the sustainable production of economically valued compounds, the production of recombinant proteins and transgenic plants of altered phenotype, and to perform metabolic engineering of plants [1,3,4,5,6,7,8]. Moreover, the enzymatic activity of the HRs may be utilized for phytoremediation and biotransformation purposes [9,10,11,12].

HR cultures usually demonstrate rapid non-geotropic growth with lateral branching in nutrient media devoid of plant growth regulators (PGRs) and with reduced concentrations of macronutrients. They are genetically stable, their biosynthetic potential is like that of the roots of the parent plant, and they do not need light to grow and synthesize natural products. However, the unsatisfactory up-scale results (from a laboratory to industrial scale) and unsatisfactory cost/profit ratio still hamper the industrial use of HRs. Plant secondary metabolites with medical applications dominated the research on natural products biosynthesized by HRs for decades. In particular, the plant metabolites currently used or with potential application in the treatment of cancer have gained a lot of interest [3,4,5,6,7,8,13,14]. Numerous compounds representing classes of plant metabolites such as coumarins, lignans, and xanthones are the subject of research not only because of their cytotoxic activity but also because of their antimicrobial, anti-inflammatory, hypoglycemic, and cardio- and neuroprotective properties [15,16,17,18,19,20,21]. A growing demand for the high-added-value molecules from plants in the face of the need to protect biodiversity may speed up the implementation of processes based on large-scale plant tissue cultures, including HRs.

In our previous reviews [22,23], attempts to improve the production of phenolic antioxidants in plant tissues using HRs have been summarized. The compounds included in the present review share their biosynthetic pathway with those discussed earlier, and some of them exhibited prominent antioxidant activities as well. Nevertheless, the currently described compounds are primarily known for their pharmacological properties other than antioxidants. The present review, based on the experimental results published before June 2025, focuses on the production of coumarins, lignans, and xanthones in HRs. The papers published in journals covered by two databases, namely, Web of Science and Scopus, were considered. The search terms used were as follows: “hairy roots”, “transformed roots”, “*Agrobacterium rhizogenes*”, or “*Rhizobium rhizogenes*” in conjunction with “coumarin”, “furanocoumarin”, “pyranocoumarin”, “osthole”, “umbelliprenin”, “lignan”, “lariciresinol”, “leptostachyol”, “phyllanthin”, “pinoresinol”, “podophyllotoxin”, “sesamin”, “xanthone”, “gambogic acid”, “mangiferin”, and “swertianin”. Research papers not containing quantitative data were excluded from the review. In contrast to numerous reviews devoted to HRs, our work puts emphasis on the quantitative aspects of the production of active natural products in vitro. Over one hundred studies that contained quantitative data have been analyzed in detail.

## 2. Coumarins, Lignans, and Xanthones in HRs

Coumarins, lignans, and xanthones are compounds that are biosynthesized in the shikimic acid pathway via the aromatic amino acids phenylalanine or tyrosine or alternatively via hydroxybenzoic acid [24,25,26,27]. Due to their physicochemical properties and diverse biological activities, the valuable plant metabolites may find use in the pharmaceutical, cosmetics, and food industries. In plants, they are implicated in the response to environmental stress and in plant–microbe interactions [28,29,30,31]. As the total chemical synthesis of these compounds is not always possible or cost-effective, alternative sources of the natural products are sought. Though HRs may offer a way to obtain these metabolites in a sustainable manner, the status of the research places them more as a tool for studying biosynthesis and its regulation.

### 2.1. Coumarins

**Figure 1:**
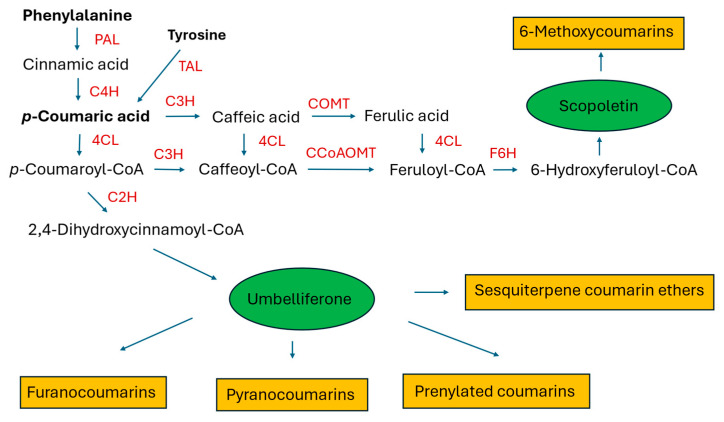
A simplified scheme of the biosynthetic pathway leading to different structural types of coumarins and the enzymes engaged in coumarin biosynthesis (key enzymes in bold): **PAL**, phenylalanine ammonia-lyase; TAL, tyrosine ammonia-lyase; **C4H**, cinnamate 4-hydroxylase; C2H, cinnamate 2-hydroxylase; C3H, p-coumarate-3-hydroxylase; **4CL**, 4-coumaroyl:CoA ligase; COMT, caffeic acid O-methyl transferase; CCoAOMT, caffeoyl-CoA O-methyl transferase; F6H, feruloyl-CoA 6-hydroxylase. Umbelliferone and scopoletin are simple coumarins that are starting points in the biosynthesis of numerous derivatives with a more complex structure.

Coumarins, like many plant polyphenols, are implicated in the plant defense response and in plant–microbiome interactions. This may be the cause of the low transformation efficiency or lack of transformation after inoculating plant material with some strains of *R. rhizogenes* (probably susceptible to the metabolites synthesized by the plant) [32,33,34]. Aside from their ecological role, coumarins are pharmacologically active compounds that demonstrate anti-inflammatory, anticonvulsant, antidepressive, anticancer, antidiabetic, antithrombotic, antioxidant, antimicrobial, osteogenic, and neuro- and hepatoprotective properties [35,36,37,38,39,40]. They are biosynthesized from shikimate via phenylalanine and p-hydroxycinnamic acid (see Figure 1) [24,25]. Overexpression of the genes encoding phenylalanine ammonia-lyase (PAL) and cinnamate 4-hydroxylase (C4H) in the HRs of Korean angelica was studied to assess whether their expression level in the plant is a limiting factor in the production of coumarins [41]. Numerous biotic and abiotic elicitors were employed to increase the accumulation of the compounds in the HRs, with various results. The quantitative data on coumarin biosynthesis in the HRs of thirteen plant species are currently available. They refer to simple coumarins, furanocoumarins, pyranocoumarins, sesquiterpene coumarin ethers, and prenylated coumarins.

#### 2.1.1. Apiaceae

**Figure 2:**
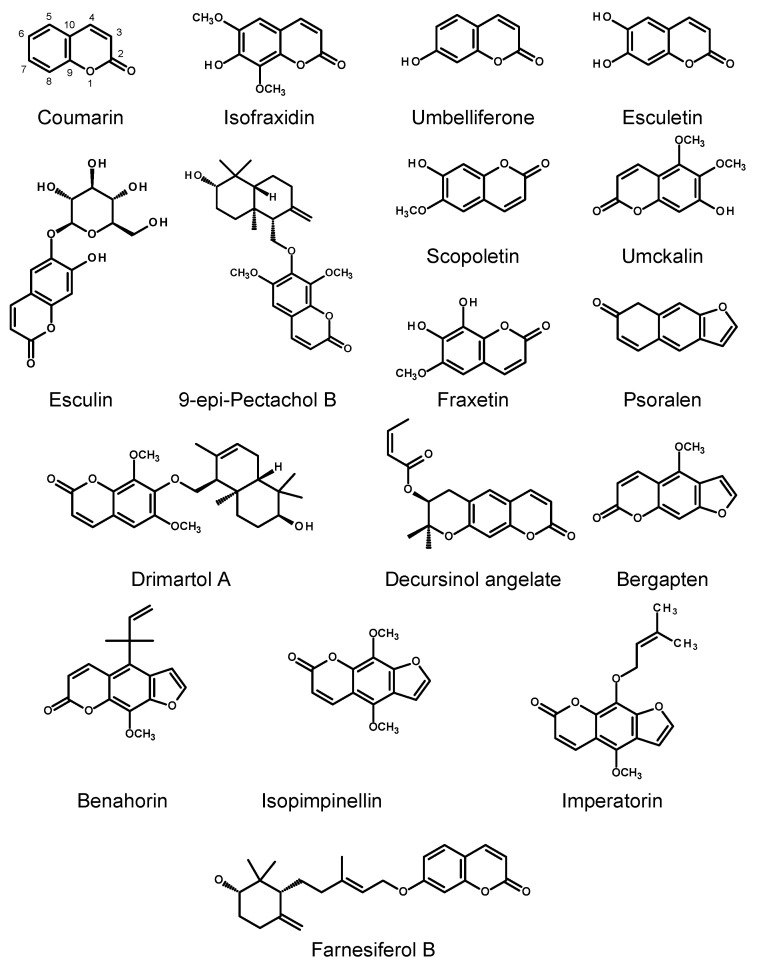
Chemical structures of selected coumarins produced by HRs. Simple coumarins (coumarin, isofraxidin, umbelliferone, esculin, esculetin, scopoletin, umckalin, fraxetin); furanocoumarins (psoralen, bergapten, benahorin, isopimpinellin, imperatorin); pyranocoumarin (decursinol angelate); sesquiterpene coumarin ethers (9-epi-pectachol B, farnesiferol B).

Królicka and coworkers [42] obtained the HRs of *Ammi majus* L. by inoculation of leaf and stalk explants from the aseptically grown plantlets with two agropine *R. rhizogenes* strains, A4 and LBA 9402. The transformation was successful only when the bacteria were treated with 200 μM of acetosyringone before inoculation, to activate *vir* gene expression. The attempts to obtain HRs with the mannopine, nopaline, and cucumopine strains proved unsuccessful. The roots showed a good growth rate (GI calculated as a ratio of the final fresh weight to the fresh weight of inoculum was 150 after 30 days of culture), but furanocoumarins characteristic of the parent plant were not found in the biomass. The only coumarin produced by the HRs was umbelliferone (19 ± 3.3 μg/g DW; for the structure, see Figure 2). Its content in the roots was similar to that found in seeds of *A. majus* and could be increased up to 32 μg/g DW by elicitation with silicon dioxide (SiO_2_). The elicitation procedures using biotic elicitors, scleroglucan and autoclaved cultures of *Erwinia chrysanthemi* or *Enterobacter sakazaki*, have proven ineffective [43].

Korean angelica (*Angelica gigas* Nakai) produces pyranocoumarins with neuroprotective and antibacterial activity. Two enzymes involved in the coumarin biosynthesis in the plant, phenylalanine ammonia-lyase (AgPAL) and cinnamate 4-hydroxylase (AgC4H), were cloned and characterized by Park and coworkers [44]. HR cultures of the plant were initiated from the leaves and stems of 30-day-old aseptic seedlings of *A. gigas* by co-cultivation with *R. rhizogenes* strain R1000 harboring either the *gus* or *AgPAL* and *AgC4H* genes under the control of a cauliflower mosaic virus (CaMV) 35S promoter [41]. HRs overexpressing the *AgPAL* gene did not demonstrate enhanced decursinol angelate (Figure 2) production when compared with the HRs expressing *gus*. The HR clones showing an elevated transcript copy number for AgC4H mRNA produced significantly higher amounts of decursinol angelate (up to 0.41 mg/g DW) than the HRs overexpressing *AgPAL* (up to 0.26 mg/g DW).

Sesquiterpene coumarin ethers, compounds with anti-inflammatory [45,46] and multidrug resistance (MDR) [47] reversing properties, are secondary metabolites frequently found in plants belonging to the Apiaceae family and the Anthemideae tribe of the Asteraceae. According to Khazaei et al. [33], 10- to 12-day-old embryo explants of *Ferula pseudalliacea* Rech. f. inoculated with *R. rhizogenes* strains ATCC 15824 or 1724 developed HRs that produced farnesiferol B, a sesquiterpene coumarin ether with anti-inflammatory activity. The accumulation of the compound in the HRs (0.75 mg/100 mg of hexane extract) was lower than that in the normal roots (1.2 mg/100 mg of extract).

#### 2.1.2. Asteraceae

HRs of *Artemisia annua* L. (Asteraceae, Anthemideae) produced the sesquiterpene coumarin ether drimartol A (up to 0.2% DW) [48]. Isofraxidin and its drimenyl ether, 9-epipectachol B, were isolated from the *R. rhizogenes* LBA 9402-transformed roots of *Tanacetum parthenium* (L.) Sch. Bip. (Asteraceae, Anthemideae) [49]. Their isolation yields were 0.012% DW and 0.014% DW, respectively. This was the first description of 9-epipectachol B as a natural compound.

Simple coumarins such as esculin, isofraxidin, and scopoletin are metabolites synthesized by many species included in the Asteraceae family. HRs of *Cichorium intybus* L. cv. Lucknow local (Asteraceae, Cichorieae) were initiated by inoculating the wounded stems of aseptic chicory seedlings with *R. rhizogenes* LMG 150 (mannopine strain) and were further cultivated in a liquid MS medium in the dark. The addition of 1.5 mM putrescine to the nutrient medium stimulated the growth of the roots (GI = 162.8 ± 12.1, calculated on a fresh weight basis after 21 days of growth) that was nearly twice as high as that of the untreated HRs. Two coumarins, esculin and esculetin, were detected in the HRs. The roots treated with 1.5 mM putrescine produced c. 39 μg/g FW esculin and c. 24 μg/g FW esculetin, whereas the untreated control roots contained c. 17 μg/g FW esculin and c. 11 μg/g FW esculetin [50]. To enhance the accumulation of coumarins in the HRs of chicory, the cultures were treated with fungal elicitors. The extracts from mycelia as well as the media filtrates from the autoclaved cultures of *Phytopthora parasitica* var. *nicotiana* or *Pythium aphanidermatum* were used for this purpose. The HRs grown in the nutrient medium supplemented with the mycelial extract of *P. aphanidermatum* (0.5–3.0%, *w*/*v*) or *P. aphanidermatum* medium filtrate (0.5% *v*/*v*) after 28 days of culture produced significantly less biomass than the control (untreated) roots. The contents of esculin and esculetin in the elicited roots were similar to those in the control roots or lower. The medium filtrate at the concentration of 1% (*v*/*v*) significantly stimulated growth of the HRs (GI = 94.5 and 76.5 in the elicited HRs and the control, respectively) and the accumulation of both esculin and esculetin (up to 85 μg/g FW and 63 μg/g FW, respectively). Both mycelial extract (0.5% and 3%, *w*/*v*) and medium filtrate (0.5–3.0%, *v*/*v*) of *P. parasitica* added to the nutrient medium enhanced the growth of the HRs (GI up to 122.4 after 28 days of culture) and production of coumarins (up to 100 μg/g FW esculin and 68 μg/g FW esculetin) [51]. Similar stimulation of growth (GI = 118.6) was observed in the HRs of chicory cultivated in medium containing 0.5 mg/L gibberelic acid (GA_3_). The roots grown in medium supplemented with 0.5 mg/L GA_3_ accumulated over 55 μg/g FW esculin and over 40 μg/g FW esculetin [52]. The paper published by the same team in 2003 [53] reported on the contents of esculin and esculetin (32 μg/g FW and 25 μg/g FW, respectively) in the HRs of chicory induced by *R. rhizogenes* LMG 150. Compared to this root culture, HRs transformed by another mannopine strain of *R. rhizogenes*, namely, A20/83, grew slower and produced less coumarin (esculin and esculetin contents: 28 μg/g FW and 23 μg/g FW, respectively). Fathi et al. [54] used cotyledonary and leaf explants of chicory to initiate HR cultures. The explants were infected with three different strains of *R. rhizogenes*: A4, ATCC 11325, and ATCC 15834 using MS medium and its modifications for the co-cultivation procedure. The KNO_3_-free MS medium stimulated the growth and branching of HRs that emerged from the cotyledonary explants infected with *R. rhizogenes* A4, whereas the modified MS medium devoid of KH_2_PO_4_ favored the production of HRs from leaf and cotyledonary explants co-cultured with *R. rhizogenes* ATCC 15834. The total phenolic content in the obtained HRs reached 4.46 mg/g DW; however, the coumarin content was not estimated.

#### 2.1.3. Rutaceae

Common rue (*Ruta graveolens* L.), known as a medicinal herb, ornamental plant, and flavoring ingredient, is a source of simple coumarins, prenylated coumarins, and furanocoumarins. The HRs of rue were initiated, with 5% efficiency, from the hypocotyls of 4-week-old axenic shoots wounded with a needle and infected using *R. rhizogenes* LBA 9402 [32]. GIs of the roots grown in a B5 [55] liquid medium without growth regulators reached 4.2 after 30 days of culture, using 1.5 g of inoculum. The GI value did not differ regardless of the light conditions. The HRs cultivated in the dark and under the photoperiod, however, differed in their coumarin content. Ten compounds, including prenylated coumarins, furanocoumarins, and dihydrofuranocoumarins, were detected in 4-week-old HRs of rue grown in the dark. Their contents ranged from 45 ± 15 μg/g DW (psoralen) to 480 ± 170 μg/g DW (bergapten). In the roots cultivated for eight weeks, an additional prenylated coumarin (benahorin, 151 ± 48 μg/g DW) and 2,5-dimethyl-7-hydroxychromon were found. The coumarin contents in the 8-week-old HRs varied from 15 ± 5 μg/g DW (psoralen) to 1060 ± 340 μg/g DW (isopimpinelin). The roots cultivated for 36 weeks contained sixteen compounds of this structural type, but their contents in the biomass significantly dropped (xanthotoxin, 2 ± 0.5 μg/g DW; rutacultin, 38 ± 12 μg/g DW; bergapten, 13 ± 4.6 μg/g DW; isopimpinelin, 13 ± 4.1 μg/g DW). The HRs cultivated under the 16 h light/8 h dark photoperiod, analyzed after four weeks of growth, accumulated eleven coumarins plus 2,5-dimethyl-7-hydroxychromon. Their contents in the roots ranged from 13 ± 4.3 μg/g DW (psoralen) to 560 ± 200 μg/g DW (bergapten). Moreover, the roots grown in the dark produced furoquinoline alkaloids (up to 390 ± 120 μg/g DW of dictamnine).

#### 2.1.4. Violaceae

An Indian medicinal plant *Hybanthus enneaspermus* (L.) F. Muell. (=*Afrohybanthus enneaspermus* (L.) Flicker), except for the macrocyclic peptides (cyclotides) that are at least partially responsible for the aphrodisiac effect of the plant [56] and flavonoids, produces coumarin (up to 2.870 ± 0.15 mg/g DW of the extract from the aerial parts) [57]. HRs of the plant were initiated from the leaf or internodal explants of both the outdoor-grown and axenic in vitro-grown shoots inoculated with different strains of *R. rhizogenes* (A4, A4T, 8196, and LBA 9402). The best root induction frequency was achieved with strain A4, and the transformed root clone A4-HRL-2B7 produced nearly three times more coumarin (0.82 ± 0.022 mg/g DW of the extract) than the roots of the outdoor-grown plants (0.28 ± 0.01 mg/g DW of the extract). However, the data provided do not address the content of this compound in the root biomass [57].

#### 2.1.5. Lamiaceae

The *R. rhizogenes*-transformed root culture of *Leonotis nepetifolia* (L.) R. Br. was initiated from the sterile 14-day-old seedlings infected with the bacterial strain A4 [58]. The LNTR4 HR clone, cultivated in liquid MS medium, was characterized by vigorous growth. After five weeks of growth, 130.75 ± 4.3 g/L (FW) and 16.3 ± 0.3 g/L (DW) of roots was obtained from 2 g of inoculum. The coumarin content was estimated at 1472.4 ± 2.563 μg/g DW of the extract and was significantly higher than that in the normal roots. Again, the presented data do not allow for the assessment of the compound content in root biomass.

#### 2.1.6. Malvaceae

*Sphaeralcea angustifolia* (Cav) G. Don, a Mexican medicinal herb used to treat fractures, inflammation, and gastric diseases, produces scopoletin, considered to be the main pharmacologically active compound. The HRs of the plant were obtained from the leaves and nodal segments of 2-month-old aseptic seedlings inoculated with the *R. rhizogenes* strains K599 (cucumopine type), A4, or ATCC 15834 (agropine type) harboring the binary vector pTDT that contained the gene for the tomato threonine deaminase enzyme, which gave a red fluorescence to the transformed plant tissue [59]. The highest transformation frequency (59.50 ± 10.5%) was achieved with ATCC 15834/pTDT strain. The HR clones obtained by the transformation with ATCC 15834/pTDT and maintained in liquid MS medium under the 16/8 h (day/night) photoperiod demonstrated different growth dynamics (GI value from 1.47 ± 0.30 to 6.84 ± 0.84 after three weeks of culture) and various levels of scopoletin accumulation (from quantities under the detection limit up to 0.151 ± 0.02 mg/g DW in 2-week-old culture of the slow-growing clone SaTR N7.2). The fastest growing HR clone (SaTR N5.1) accumulated 0.004 ± 0.002 mg/g DW of scopoletin, after three weeks of culture. The compound was also secreted into the culture medium (up to 0.73 ± 0.44 mg/L). In the scopoletin accumulating HRs cultivated for two years, the content of the compound ranged from 0.001 ± 0.0003 to 0.011 ± 0.002 mg/g DW.

The roots of *Urena lobata* L. contain a furocumarin, imperatorin, with anti-inflammatory activity and cytotoxicity towards several cancer cell lines. The HRs of *U. lobata* emerged from leaf explants excised from 15-day-old aseptic seedlings and infected with *R. rhizogenes* strain ATCC 15834 [60]. The roots were cultivated in liquid Woody Plant Medium (WPM) [61] in the dark. Six groups of the obtained HR clones (a total of 30 clones) were characterized in detail. The roots differed in their growth rate (GI calculated on a dry weight basis ranged from 13.0 ± 0.7 to 54.3 ± 1.8 after 35 days in culture), morphology, and imperatorin content (up to 0.14 μg/g DW) [60]. The addition of chitosan to the nutrient medium (10 mg/L) markedly increased imperatorin production in the HRs (498.29 μg per flask in the treated roots versus 13.54 μg per flask in the controls) and did not affect the growth of roots [62]. A cryopreservation procedure for highly productive HR clones of *U. lobata* has been developed as well [63].

#### 2.1.7. Fabaceae

The HRs of *Melilotus albus* Medik. were obtained by the infection of healthy *M. albus* seedlings with *R. rhizogenes* strain K599 harboring the OE-MaCYP82L1 (*MaCYP82L1* gene connected to plant overexpression vector pH7FWG2-RR) [64]. The transgenic roots contained 0.17‰, 0.23‰, and 0.24‰ (170–240 μg/g) of coumarin, which was 2–3-fold more than in the control proving that MaCYP82L1, an important regulator that adjusts drought and salt resistance, is implicated in coumarin metabolism.

#### 2.1.8. Geraniaceae

The South African medicinal plant *Pelargonium sidoides* DC, whose tubers are valued as a raw material to produce drugs effective in the treatment of bronchitis, synthesizes highly oxygenated simple coumarins [65]. To initiate HR cultures, 7-day-old *P. sidoides* seedlings and two strains of *R. rhizogenes* were used (A4T and LBA 9402) [66]. The roots derived from the bacterial transformation (five AT4 and two LBA 9402 clones) were cultivated in a half-strength liquid MS medium in the dark, giving 1.2–4.5 g FW per flask after 28 days in culture (inoculum: 0.002–0.006 g FW). The most productive HR clones accumulated 5.79–8.82 μg/g DW of scopoletin and 6.25–8.20 μg/g DW of fraxetin. The contents of the two coumarins in the bulbs of the intact plant were estimated at 6.09 and 2.34 μg/g DW, respectively. Yousefian et al. [67] obtained HRs of *P. sidoides* by inoculation of the leaf explants (excised from sterile seedlings) with *R. tumefaciens* C58C1 (*pRiA4*) and found that the LBA 9402 strain was not effective for HR induction. Cultures initiated by the inoculation of 0.5 g FW of roots on a solidified MS medium, after four weeks of culture in the dark, produced 4.0–9.6 g FW and 0.24–0.59 g DW of biomass, depending on the root clone. The agitated liquid cultures in MS medium (50 mL) produced up to 22.61 g/flask (FW) on day 35 of culture (inoculum: 2 g FW) [68]. Upon elicitation with methyl jasmonate (MJ; 100 μM), the HRs produced up to 427 μg/g DW of umckalin (see Figure 2) [67]. The result, however, should be interpreted with caution due to the quality of the chromatogram presented and inconsistencies between the text and the figures included in the paper. The total phenolics content (TPC) in HRs of *P. sidoides* reached 475.66 ± 37.61 mg GAE/g FW in one of the investigated root clones, harvested after 14 days of culture [68].

The HR cultures that accumulated coumarins of potential use in medicine and cosmetics are presented in Table 1.

### 2.2. Lignans and Neolignans

**Figure 3:**
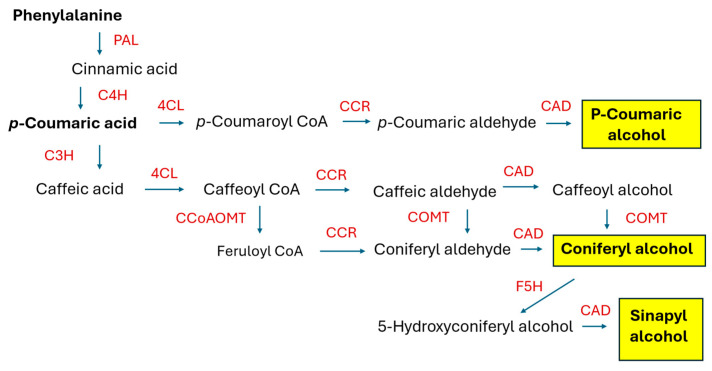
The biosynthetic pathway of monolignols (in yellow rectangles). PAL, phenylalanine ammonia-lyase; C4H, cinnamate 4-hydroxylase; 4CL, 4-coumaroyl:CoA ligase; C3H, p-coumarate 3-hydroxylase; CCoAOMT, caffeoyl-CoA O-methyl transferase; CCR, cinnamoyl-CoA reductase; CAD, cinnamyl alcohol de-hydrogenase, COMT, caffeic acid O-methyl transferase; F5H, ferulate 5-hydroxylase. PAL, C4H, and 4CL are considered key enzymes in the phenylpropanoid pathway, and their activity is often monitored in experiments using HRs.

This group of secondary (specialized) metabolites is commonly found in plants. Nevertheless, the most attention is paid to the lignans of potential application in cancer treatment and to the lignans that occur in food plants and are a component of the human diet [18,69,70,71]. As the podophyllotoxin derivatives such as teniposide and etoposide have found clinical application, new plant sources of aryltetralin lignans, including plant in vitro cultures, have been intensively sought. The total synthesis of lignans encounters obstacles such as the specific stereochemical configuration of these compounds. Though the biosynthesis of monolignols in plants is well understood (see Figure 3), the complete biosynthetic routes leading to the individual compounds are still being investigated. HRs are a useful tool in this research.

#### 2.2.1. Asteraceae

**Figure 4:**
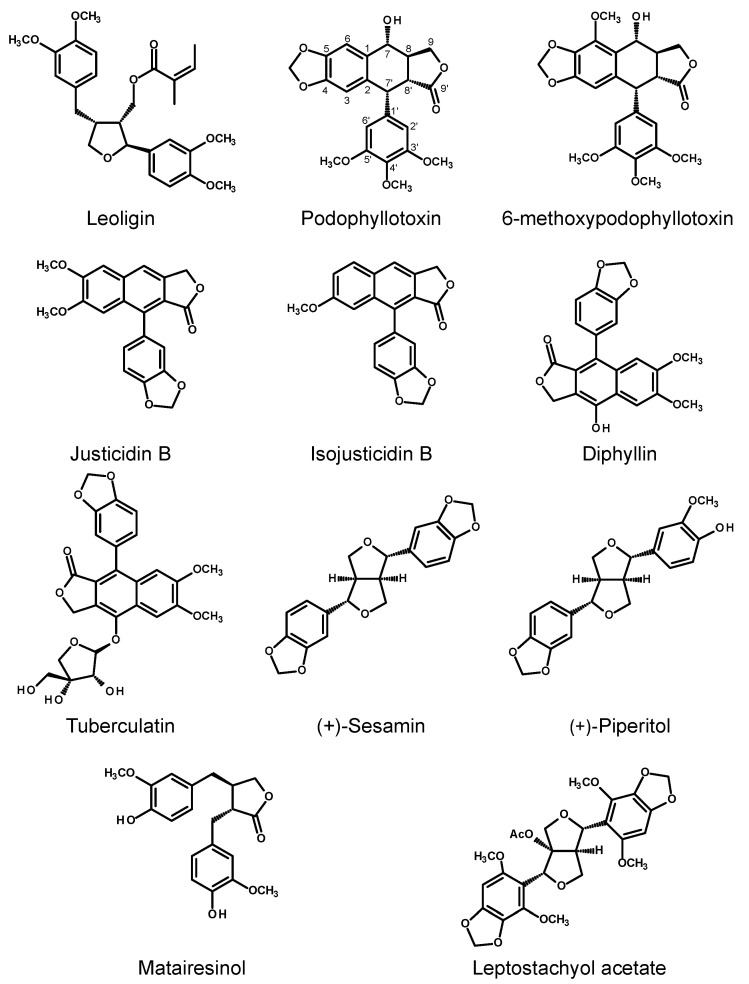
Chemical structures of selected lignans produced by the HRs of *Leontopodium nivale* ssp. *alpinum* (leoligin), *Linum* spp. (podophyllotoxin, 6-methoxypodophyllotoxin, justicidin B, isojusticidin B, diphyllin, tuberculatin, matairesinol), *Sesamum indicum* (sesamin, piperitol), and *Phryma leptostachya* (leptostachyol acetate).

Wawrosch et al. [72] established an HR culture of *Leontopodium nivale* ssp. *alpinum* (Cass.) Greuter (Asteraceae, Gnaphalieae), known as alpine edelweiss, via infection of the leaves of aseptically grown shoots with *R. rhizogenes* strain ATCC 15834. The obtained HRs were cultivated in a liquid MS medium containing 3% sucrose in the dark and were subcultured every four weeks (0.5 g of inoculum, 50 mL of nutrient medium). The final dry weight of a fast-growing line of the HRs (K8A) was 0.62 ± 0.03 g after a 4-week culture. The roots accumulated lignans, including leoligin (0.0062 ± 0.0021% DW), which activates cholesteryl ester transfer protein and reduces LDL cholesterol levels [73,74,75] (for the structure, see Figure 4), and its derivative 5-methoxy-leoligin (0.0049 ± 0.0014). Silver nitrate (AgNO_3_; 15, 30, and 60 μM), sucrose (5, 6, and 7%), yeast extract (YE; 1, 2, and 5 g/L), and MJ (50, 100, 200, and 300 μM) were used to enhance the productivity of the culture. Sucrose, at a concentration of 6%, significantly enhanced the accumulation of leoligin (0.0678 ± 0.0042% DW) and its derivative (0.0372 ± 0.0025% DW) without significant impacts on the growth of the HRs. Likewise, the addition of 2 g/L YE to the nutrient medium did not affect the growth of the roots and increased the accumulation of lignans (0.0337 ± 0.0094% DW and 0.0204 ± 0.0050% DW, respectively). The remaining elicitors, although they increased the contents of lignans in the HRs, hindered the growth of roots.

A new, formerly undescribed neolignan was isolated from the HRs of *Cichorium intybus* grown in a modified MS liquid medium containing half-strength macronutrients and using a 16/8 h photoperiod. Its chemical structure was established as (7S, 8*R*)-3′-Demethyl-dehydrodiconiferyl alcohol-3′-*O-β*-glucopyranoside. Judging by the isolation yield of the compound, its content in the root biomass was at least 0.12 mg/g DW [76].

#### 2.2.2. Brassicaceae

**Figure 5:**
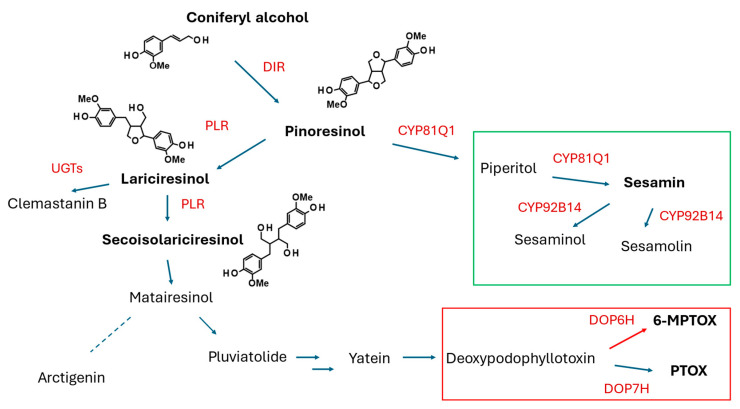
A simplified scheme of the lignan biosynthesis pathway showing the route of synthesis of furofuran lignans (pinoresinol, piperitol, sesamin, sesamolin, and others), tetrahydrofuran lignans (lariciresinol, clemastanin B), dibenzylbutane lignans (secoisolariciresinol), dibenzylbutyrolactone lignans (matairesinol, arctigenin, pluviatolide, yatein), and aryltetralin lignans (justicidin B, podophyllotoxin, and its derivatives). DIR, dirigent protein; PLR, pinoresinol reductase; UGT, glucosyltransferase; DOP6H, deoxypodophyllotoxin-6-hydroxylase; DOP7H, deoxypodophyllotoxin-7-hydroxylase.

Lariciresinol (LARI, see Figure 5) and its derivatives play a pivotal role in the antiviral activity of *Isatis indigotica* Fortune (Brassicaceae, Isatideae), a Chinese medicinal plant [77]. Roots of the plant (*Radix Isatidis*), also known as Ban-Lan-Gen in Chinese, have been traditionally used as a remedy for influenza, viral pneumonia, mumps, pharyngitis, and hepatitis. Laricinol molecules are synthesized starting from the monolignol coniferyl alcohol (see Figure 5), which is dimerized to form a furofuran lignan, pinoresinol (PINO), by a dirigent protein (DIR). The lignan is further processed by pinoresinol/lariciresinol reductase (PLR) to produce lariciresinol [78].

The *I. indigotica* HR culture, used for studies on lariciresinol biosynthesis, was obtained via the infection of sterile plantlets with the *R. rhizogenes* strain C58C1. The culture was maintained in Erlenmeyer flasks (250 mL) filled with 100 mL of a liquid half B5 medium each in the dark [79]. An analysis of the RNA sequencing (RNA-Seq) expression profile and lignan (pinoresinol, lariciresinol, secoisolariciresinol, mataireisnol, secoisolariciresinol diglucoside) contents in *I. indigotica* HRs elicited with 0.5 μM MJ suggested that *I. indigotica* pinoresinol/lariciresinol reductase 1 (*Ii*PLR1) is implicated in lariciresinol accumulation in the roots. Suppression of the *IiPLR1*, *IiPLR2*, and *IiPLR3* genes in the roots and subsequent analysis of the lariciresinol content in the HRs supported the assumption that *Ii*PLR1 is the only enzyme of the *Ii*PLR members that influences lignan accumulation. HRs overexpressing *IiPLR1* demonstrated significantly enhanced lariciresinol accumulation (up to 353.9 μg/g DW), which was c. 6.3-fold increased when compared to the wild-type HRs, whereas the production of biomass remained unaltered (c. 8 g FW/flask) [78]. A similar model was used to study the role of *IiC3H* (gene encoding coumarate 3-hydroxylase), identified as a key gene in the lariciresinol biosynthetic pathway in *I. indigotica* [80]. HRs of *I. indigotica* with overexpression of *IiC3H* were obtained by the infection of sterile seedlings with disarmed *R. tumefaciens* strain C58C1 harboring both the *R. rhizogenes* Ri plasmid pRiA4 and the plasmid pCAMBIA1304-IiC3H containing the full-length *IiC3H*. The regenerated HRs were cultivated in a half MS liquid medium in the dark. The lignan content analysis demonstrated that using this approach, the accumulation of lariciresinol could be increased up to 94 mg/g DW (23.8 mg/g DW in control). The authors, however, did not comment on the growth parameters of the engineered HRs. The same research team confirmed that the enhanced activity of PAL, induced by elicitation with 100 μM MJ, correlated with the increased accumulation of lariciresinol in the HRs of *I. indigotica* [81] and revealed that in the lariciresinol biosynthetic pathway in this plant, 4CL3, a member of the 4CL (4-coumaroyl:CoA ligase) protein family, which demonstrated the highest affinity to caffeic acid, plays a special role [82]. Further studies on the lignan biosynthesis in *I. indigotica* were conducted using HRs of the plant as a tool to investigate the functions of the individual enzymes and transcription factors. A transcription factor from the AP2/ERF family, *Ii049*, turned out to be a positive regulator of lignan biosynthesis in *I. indigotica*. Furthermore, the transcription of the SA biosynthesis genes was also regulated by the factor. Because SA could induce the accumulation of lignan and the expression of lignan/lignin biosynthetic pathway genes, *Ii049* may control lignan biosynthesis by regulating the genes involved in lignan/lignin biosynthesis and by regulating SA biosynthesis [83]. Lariciresinol-4-*O*-*β*-D-glucoside and clemastanin B (lariciresinol-4,4′-bis-*O*-*β*-D-glucoside) synthesized by *I. indigotica* have antiviral activity. Several candidate genes, encoding UDP-glucose-dependent glycosyltransferases, were either silenced or overexpressed in HRs of the plant to clarify their role in the biosynthesis of the individual lariciresinol glycosidic derivatives. Overexpression of *IiUGT1* and *IiUGT71B5a* in the HRs significantly enhanced the accumulation of pinoresinol diglucoside, lariciresinol-4-*O*-*β*-D-glucoside, lariciresinol-4′-*O*-*β*-D-glucoside, and clemastanin B in the roots, unlike *IiUGT4,* which did not affect the pinoresinol diglucoside content [84].

#### 2.2.3. Linaceae

Aryltetralin lignans from *Linum* spp., including podophyllotoxin and its derivatives, are of interest due to their use in cancer therapy [85]. Moreover, flax seeds, as a component of the human diet, are an important source of lignans with potentially beneficial effects on human health [86]. Attempts to obtain these active compounds from the in vitro cultures of *Linum* spp. were summarized in 2014 by Malik et al. [87] and in 2024 by Nedelcheva et al. [88].

Studies on aryltetralin lignan biosynthesis in HRs of *Linum* were initiated in 1993 by Oostdam and coworkers [89]. Leaf explants of *L. flavum* L. were infected with three *R. rhizogenes* strains with different virulence levels: LBA 9402, 8490, and 9365. HRs emerged only from the explants inoculated with strain LBA 9402. The HRs were grown in a liquid MS medium supplemented with B5 vitamins and 2% sucrose in the dark and were subcultured monthly. The HPLC and GC-MS analyses confirmed that the transformed roots contained 6-methoxypodophyllotoxin (6-MPTOX; in the original paper named 5-methoxypodophyllotoxin according to the different carbon atom enumeration system applied) and trace amounts of *α*- and *β*-peltatin. The 6-MPTOX content ranged from 1.4 to 3.5% DW, depending on the analyzed root clone. The production of 6-MPTOX reached 1.4 mg/g DW per day. Due to the low degree of lateral branching, the doubling time of the cultures was longer than expected (11–29 days). Later, the HRs of several *Linum* species were investigated in a search for a highly productive source of the pharmacologically active lignans. The HRs obtained by the transformation of *L. strictum* ssp. *strictum* L. with *R. rhizogenes* TR 105 and grown in a liquid MS medium in the dark produced 6-MPTOX. However, the content of this metabolite in the biomass was low (0.57 ± 0.09 mg/g DW) [90]. Hinokinin was the major lignan found in the HRs of *L. corymbulosum* Raichenb. maintained in MCW medium (0.11–0.17 mg/g DW). Suspension cultures of the species accumulated c. 0.05 mg/g DW after 22 days of growth [91]. The HRs of *L. tauricum* Willd. initiated by inoculation with *R. rhizogenes* strain ATCC 13834, synthesized mainly 4′-demethyl-6-MPTOX and 6-MPTOX (25.92 ± 0.31 mg/g DW and 35.06 ± 0.03 mg/g DW, respectively). Elicitation of the culture with 150 μM MJ (7 days of treatment) increased the accumulation of 4′-demethyl-6-MPTOX up to 31.92 ± 0.15 mg/g DW and 6-MPTOX up to 36.19 ± 0.08 mg/g DW [92].

Leaf explants inoculated with *R. rhizogenes* strain ATCC 15834 gave rise to the HR culture of *L. lewisii* Pursh. Concomitantly, the adventitious root culture of the species was developed. The cultures were examined with respect to the growth kinetics and production of the aryltetralin lignans [93]. The growth performance of the HRs was better than that of the adventitious roots. The HRs produced c. 3.5 g of fresh biomass and 4.77 ± 0.50 mg of justicidin B, after 5 weeks of growth, whereas the adventitious root culture gave c. 2.5 g of roots and 3.43 ± 0.14 mg of the lignan. Except for justicidin B with cytotoxic and antiviral properties, isojusticidin B, traces of secoisolariciresinol, tuberculatin, and diphyllin glycosides were also found in the roots. The three-week-old cultures were treated with MJ (100 μM), coronatine (1 μM), and SA (50 and 100 μM) or fed with ferulic acid (1 mM) for 7 days. Coronatin alone or together with ferulic acid, as well as MJ applied together with ferulic acid were the most effective treatments for the stimulation of justicidin B production. Its content in the HRs reached over 40 mg/g DW. The HRs cultured for 4 weeks in a 1 L stirred tank bioreactor produced 82.2 mg/L of justicidin B. The amount could be increased up to 204.1 mg/L by the treatment with MJ.

HRs initiated by the direct incubation of segments excised from the sterile grown plantlets of *L. austriacum* L. with *R. rhizogenes* LBA 9402 and cultivated in a liquid half B5 medium in the dark produced justicidin B (16.9 mg/g DW) and its isomer isojusticidin B (2.5 mg/g DW after 30 days in culture). The productivity of *L. austriacum* HRs was significantly higher than those of the cell suspension and calli of the plant [94]. According to Mohagheghzadeh and coworkers, the HRs of *L. austriacum* obtained with *R. rhizogenes* ATCC 15834 and grown in cotton bags submerged in the liquid WPM medium in the dark accumulated neither PTOX nor 6-MPTOX (the justicidin B content was not examined). Their co-culture with the HRs of *L. persicum* Ky. ex Boiss. did not trigger PTOX biosynthesis. However, the HRs of *L. persicum* cultivated alone contained 0.02 ± 0.01 g/100 g DW PTOX and 0.17 ± 0.01 g/100 g 6-MPTOX (GI of the 60-day-old culture: 7.18 ± 0.45) [95]. Three in vitro culture systems, callus culture, adventitious roots culture, and HRs of *L. austriacum* (induced by inoculation with *R. rhizogenes* ATCC 15834), were assessed with respect to their productivity [96]. The adventitious roots and the HRs demonstrated similar growth kinetics, reaching c. 4 g FW at the end of the 4-week growth cycle. Cell cultures reached c. 3 g FW after two weeks of growth. The total phenolic content was the highest in the HRs. Justicidin B was undetectable in cell cultures, but adventitious roots and HRs accumulated similar amounts of the compound (4–5 mg/g DW). After the elicitation with 100 μM MJ or 10 μM coronatine (96 h), cell cultures started to synthesize justicidin B, albeit in small amounts, and the production of the lignan in root cultures was significantly enhanced. Coronatin was more effective in the adventitious root culture (justicidin B content: 15.74 ± 1.18 mg/g DW), while the HRs responded better to MJ treatment (14.71 ± 0.90 mg/g DW). One of the examined HR lines was chosen to initiate a culture in the 1 L stirred tank bioreactor. The culture demonstrated a 5-fold increase in the biomass after 21 days of growth and produced 21 mg/g DW of justicidin B. After a longer growth period (35 days) in the bioreactor, the HRs reached a 14-fold increase in biomass but accumulated only 9 mg/g DW of the lignan.

Justicidin B was also the major metabolite synthesized by HRs of *L. leonii* F.W. Schultz., derived from the segments of sterile grown plants incubated with *R. rhizogenes* strain ATCC 15834 [97]. The HRs accumulated up to 10.8 mg/g DW of the lignan, five times more than the callus cultures of *L. leonii*. The same culture grown in a bioreactor (2 L, inoculum: 3 g) for 40 days produced 27.2 g DW/L of the root biomass and 1.55 ± 0.07% DW of justicidin B [98]. From the HRs of *L. leonii* maintained in MS medium supplemented with 1 g/L casein hydrolysate in the dark and subcultured every 21 days, five more aryltetralin lignans were isolated: diphyllin, tuberculatin, phyllantusmin C, reticulatuside A, and cilinaphthalide C. Moreover, two norditerpenoids, one of the podocarpane type (nimbinone) and one of the isopisiferin group of icetaxane terpenoids (leonsiferon), were found in the roots [99].

The biosynthesis of justicidin B in *L. perenne* Himmelszelt was investigated using the HRs obtained by cocultivation of plant explants with genetically engineered or wild-type *R. rhizogenes* strain TR105. The HRs with silenced (+)-pinoresinol–(−)-lariciresinol reductase (PLR) encoding gene (*PLR-Lp1*) accumulated less justicidin B (up to 11 mg/g DW) than the HRs without any construct (up to 37 mg/g DW in 14 days), thus confirming the participation of PLR in justicidin B biosynthesis [100]. The HRs of *L. perenne* L. which emerged after the infection of sterile seedling explants with *R. rhizogenes* ATCC 15834, produced 20 g/100 mL of fresh biomass after 18 days of culture. The roots contained justicidin B, isojusticidin, diphyllin and its glycosides, and two diastereoisomers of 6-MPTOX [101].

A mikimopine wild strain A13 of *R. rhizogenes* was used to induce HR culture of *L. mucronatum* ssp. *mucronatum*. The HRs, grown in a liquid MS medium in the dark, produced up to 5.55 ± 0.76 mg/g DW of PTOX, 41.38 ± 1.74 mg/g DW of 6-MPTOX, and nearly 6 g per flask fresh biomass after 56 days of growth [102,103].

An effect of elicitation on growth and lignan accumulation in *L. album* Kotschy ex Boiss. HR culture was investigated using HRs derived from the aseptic cotyledons inoculated with *R. rhizogenes* LBA 9402 and cultivated in a GB5 medium under the 16/8 h (day/night) photoperiod [104]. An autoclaved or filter-sterilized culture filtrate of *Piriformospora indica* (a fungal endophyte), at a concentration range of 0.5–5% (*v*/*v*), was used as an elicitor. Addition of the filter-sterilized *P. indica* culture filtrate (3%) to 10–12-day-old HRs and 48–96 h of treatment were optimum for increasing the productivity of the culture. The elicited HRs produced from 18.2 to 20.9 g DW/L of biomass, 225.7–233.8 mg/L PTOX, and 94.5–131.9 mg/L 6-MPTOX. The enhanced accumulation of lignans was accompanied by the high activity of PAL. The control roots produced 60.9 ± 3.1 mg/L PTOX, 29.7 ± 1.0 mg/L 6-MPTOX, and 15.6 ± 0.5 g DW/L of biomass.

Tashackori and coworkers published a series of studies regarding the elicitation of the *L. album* HRs with preparations of *P. indica* [105,106,107,108]. The HRs (transformed with *R. rhizogenes* LBA 9402) grown in MS medium in the dark and subcultured every three weeks, were treated with the *P. indica* mycelial extract (1%, *v*/*v*) for 12, 24, 48, 72, and 120 h. The fungal extract was added to 10-day-old HR cultures. The DW of elicited roots transiently increased in comparison with the control HRs after 24 h of treatment. After 120 h, the DW of the elicited roots did not differ from that of the control. The maximum lignan content was measured 24 h after the addition of the elicitor (91.019 μg/g DW PTOX; 157.3 μg/g DW LARI; and 13 mg/g DW 6-MPTOX). The activity of PAL correlated with the accumulation of lignans. Starting from 72 h of treatment, significant differences in the lignan contents and DW of roots between the elicited and control HRs were not observed [105]. A cell wall preparation from *P. indica* (0.5, 1.0, 2.5, and 5%; *v*/*v*) added to the 10-day-old *L. album* HRs significantly and dose-dependently reduced the growth of the roots after 5 days of treatment. The elicitor at a concentration of 1% after 5 days of treatment significantly increased the contents of lignans in the HRs (124.18 μg/g DW LARI; 124.46 μg/g DW PTOX; 57.52 μg/g DW PINO; 12.58 mg/g DW 6-MPTOX). The higher contents of lignans, however, did not compensate for the reduced growth. The time course experiment revealed that the elicitor (1%) markedly enhanced the accumulation of lignans starting from 24 h (PINO, PTOX) and 48 h (LARI, 6-MPTOX) after the addition of the fungal preparation. The accumulation of phenolic acids (cinnamic, coumaric, caffeic, ferulic, and salicylic) was significantly increased after just 12 h of treatment. The PAL and CAD activities were significantly higher 48, 72, and 120 h after addition of the elicitor. Maximum expression of the *PAL* and *CCR* genes took place 48 h after adding the fungal preparation. Maximum expression of *CAD* and *PLR* occurred earlier (24 h) [106]. The consequences of silencing the PINO/LAR reductase encoding gene (*PLR*) in the HRs were investigated in the next series of experiments [107]. In the genetically engineered roots, the PLR activity and contents of aryltetralin lignans (PTOX, 6-MPTOX) were significantly reduced. A tendency toward the reduction of root growth was observed as well. On the contrary, the contents of PINO, LARI, phenolic acids, and amino acids were significantly elevated in the HRs with silenced PLR. The elicitation experiments with the HRs harboring the silenced PLR proved that the elevation of the PINO and LARI contents did not lead to the enhanced accumulation of PTOX and 6-PTOX but resulted in the accumulation of lignin. In the elicited HRs with silenced *PLR*, the contents of phenolic acids and amino acids were higher than those in the control roots. In the following experiment, a chitinase-treated cell wall preparation of *P. indica* (1%, *v*/*v*) was added to the culture medium (30 mL) in which 2 g of the *L. album* HRs was placed. Different parameters of the culture were followed: H_2_O_2_ production; activities of PAL, CAD, superoxide dismutase (SOD), and glutathione peroxidase (GPX); contents of lignans, lignin, phenolic acids, amino acids, and flavonoids; and expression of *PAL*, *CAD*, *CCR,* and *PLR*. Measurements were carried out 12, 24, 48, 72, and 120 h after the initiation of the experiment. After just 12 h, the roots responded to the treatment with elevated H_2_O_2_ generation, and the activities of SOD and GPX were higher than those in control cultures. After 24 h, the contents of PINO, LARI, and PTOX in the treated roots began to exceed those observed in the controls. This was accompanied by higher activities of PAL and CAD. After 72 h, enhanced accumulation of 6-MPTOX could be observed in the elicited roots. The contents of phenolic acids, lignin, and some flavonoids also increased in the HRs treated with the fungal preparation [108].

Two clones of HRs were obtained by inoculation of the young *L. album* stems with *R. rhizogenes* strain LBA 9402 and *R. tumefaciens* C58C1 (pRiA4). The HRs (inoculum: 2.4 g FW) were grown in MS medium (50 mL) for four weeks. The clone induced by strain LBA 9402 demonstrated better growth (12.5 g FW after four weeks of growth) and higher lignan contents (105 μg/g DW PTOX; 48 mg/g DW 6-MPTOX) than those in the HRs induced by *R. tumefaciens* C58C1 (pRiA4) [109]. The HRs obtained by the inoculation with the *R. rhizogenes* strain LBA 9402 were treated with various biotic elicitors: chitosan and three fungal extracts (*Fusarium graminearum*, *Sclerotinia sclerotiorum*, and *Trichoderma viride*). Elicitors were added after 12 days of growth, and the HRs were cultivated for another 6 days. The treatments did not significantly affect the growth of the HRs. Maximum lignan contents were observed six days after the addition of elicitors. The extract from *F. graminearum* was the most effective in increasing the production of LARI (260 μg/g DW) and PTOX (up to 200 μg/g DW). The best stimulation of 6-MPTOX production (up to 160 μg/g DW) was observed in the HRs treated with *T. viride* [110].

Cong et al. [111] monitored changes in the aryltetralin lignan profile in each growth phase of *L. album* and *L. flavum* HRs. Only two strains of *R. rhizogenes*, LBA 9402 and ATCC 15834, were able to initiate the development of HRs from hypocotyl explants of the two mentioned *Linum* species. The root clones that demonstrated the fastest growth were chosen for the experiments. The HRs (2 g FW) were inoculated in 100 mL of a liquid GB5 medium and maintained for three weeks under the 16/8 h (light/dark) photoperiod. At the end of the culture cycle, the final FW of the HRs was 5.6 g and 19.4 for *L. album* and *L. flavum*, respectively. The HRs of *L. album* entered the stationary phase of growth after 17 days of culture, whereas the HRs of *L. flavum* reached this phase of growth already 13 days after inoculation in the fresh medium. Ten aryltetralin lignans were found in the cultures: PTOX, *β*-peltatin, 6-MPTOX, PTOX glucoside, peltatin glucoside, 6-MPTOX glucoside, 4′demethyl-deoxyPTOX glucoside, 5′demethoxy-6-MPTOX, 5′demethoxy-6-MPTOX glucoside, and 4′demethyl-6-MPTOX glucoside. Except for PTOX, glucosylated forms of the lignans were detected mostly in the stationary phase of culture. The glucosylated form of PTOX was found exclusively in the HRs of *L. album*; however, its content was much lower than that of the aglycon. The HRs produced 3.38 μM/flask (1.4 mg/flask; *L. album*) and 0.62 μM/flask (0.26 mg/flask; *L. flavum*) PTOX and 77.62 μM/flask (34.5 mg/flask; *L. album*) 6-MPTOX or 308.64 μM/flask (137.08 mg/flask; *L. flavum*) 6-MPTOX plus its glucoside. Correlation networks of aryltetralin lignans in *L. album* and *L. flavum* and the balance between the lignans and their glucosylated forms were described [111].

Feeding experiments using coniferylaldehyde as a precursor of the lignans were conducted in HRs of *L. album* [112]. As a result, an enhanced accumulation of LARI (up to 107.61 μg/g FW), PINO (up to 8.7 μg/g FW), and PTOX (6.42 μg/g FW) in the roots fed with 2 mM/L coniferylaldehyde was observed.

Lalaleo and coworkers studied four kinds of *L. album* in vitro cultures, namely, cell suspension culture, adventitious root culture, HR culture (transformed with *R. rhizogenes* LBA 9402), and transformed cell suspension obtained from the HRs, in terms of the production of biologically active lignans [113]. The in vitro-grown *L. album* plantlets as well as both types of cell suspensions preferentially accumulated PTOX (up to 47 μg/g DW in the non-transformed cell suspension). The root cultures produced mainly 6-MPTOX (up to 15 mg/g DW in the adventitious roots and up to 9.5 mg/g DW in the HRs). Elicitation with 1 μM coronatine increased the expression of *LaCCR* and *LaCAD* in the non-transformed cell suspension and the expression of *LaPLR* in the transformed cells. The higher expression levels of the genes, however, did not result in increased lignan accumulation. The elicited adventitious roots demonstrated higher expression of *LaCCR* and *LaPLR* and transiently enhanced (by approximately 20%) 6-MPTOX accumulation. The HRs upon elicitation with coronatine showed increased expression of *LaCCR*, *LaCAD*, and *LaPLR* but did not produce more 6-MPTOX.

The possible role of H_2_S as a regulatory molecule engaged in the modulation of root metabolism was investigated by Fakhari et al. [114]. The HRs of *L. album*, 11 days after inoculation (2 g FW) in a fresh liquid MS medium (30 mL), were treated with 0.5 and 1.0 mM NaHS (as a H_2_S donor). The treated HRs demonstrated diminished biomass accumulation (7.6–10.8% in comparison with the controls); enhanced activities of the antioxidant enzymes (SOD, catalase); transiently increased production of H_2_O_2_ and NO; diminished contents of caffeic, coumaric, and ferulic acids; and enhanced accumulation of PTOX (up to 135 μg/g DW) and 6-MPTOX (up to 30 mg/g DW). Elicitation with 200 mg/L chitosan induced, to some extent, similar effects in the HRs [115]. The contents of H_2_O_2_ and malonyl dialdehyde (MDA), markers of oxidative stress, as well as the NO production, increased transiently just 12 h after adding chitosan, and concomitantly, the antioxidative enzymes (SOD, catalase, GPX) were activated in the elicited HRs. Changes in the pool of free and conjugated polyamines were also observed. The elicitation enhanced the activity of PAL and tyrosine ammonia-lyase (TAL) and the contents of phenolic acids (cinnamic, coumaric, caffeic, ferulic, and salicylic). The accumulation of PTOX and 6-MPTOX in the HRs increased and reached a maximum 72 h after the addition of chitosan (146.20 ± 2.27 μg/g DW and 39.04 ± 0.73 mg/g DW, respectively). The content of LARI was transiently increased after 24 h of treatment. The HRs treated with 0.25 mM putrescine responded likewise [116]. The contents of PTOX and 6-MPTOX reached maximum levels 120 h after the addition of putrescine (c. 150 μg/g DW and c. 80 mg/g DW, respectively).

The HRs of *L. flavum* were initiated from hypocotyls of the seedlings by inoculation with *R. rhizogenes* strains LBA 9402 and ATCC 15834. A dozen of the HR clones were examined for their growth kinetics and lignan contents. The GI values of the HRs grown in a GB5 medium ranged from 3.4 to 5.9, after 20 days of culture. The roots produced mainly 6-MPTOX glucoside (4.1–24.1 mg/g DW) and 6-MPTOX (1.5–8.4 mg/g DW). PTOX glucoside (1.2–4.9 mg/g DW) and PTOX (0.2–0.8 mg/g DW) were accumulated in smaller quantities [117]. One of the HR clones, characterized by both good productivity and a stable phenotype, was chosen for further studies. The GB5 medium and 14-day culture cycle were optimum for the selected HRs. 6-MPTOX glucoside was found not only in the roots (over 20 mg/g DW) but was also present in the nutrient medium (nearly 2 mg/mL), which is important for technological reasons. 6-MPTOX in its free form was accumulated in the roots (over 10 mg/g DW) and was excreted into the medium (less than 0.5 mg/mL). The increased sucrose content in the nutrient medium (6%) stimulated the production of both biomass (25.1 g FW per flask) and aryltetralin lignans (intracellular 6-MPTOX glucoside: over 50 mg/g DW; extracellular 6-MPTOX glucoside: over 5 mg/g DW). The total 6-MPTOX content in the HRs (in free and glucosylated forms) was 6.5% DW. Elicitation experiments using abscisic acid, MJ, SA, and YE revealed that the elicitors enhanced the excretion of the lignans into the culture medium and that each of them modified the lignan biosynthesis in a specific way. The treatment with MJ (100 μM) increased the accumulation of 6-MPTOX (total 7.5% after 48 h) but negatively affected the growth of the roots. Feeding with L-phenylalanine (1 mM) or ferulic acid (1 mM) demonstrated that the HRs fed with ferulic acid, after 48–96 h of treatment, produced more PTOX and 6-MPTOX than the control roots and the roots fed with L-phenylalanine or treated with Tween-20 (2% *w*/*v*). Tween-20 as a permeabilizing agent enhanced the liberation of lignans into the culture medium but did not stimulate their production.

Flax (*L. usitatissimum* L.) is a rich dietary source of (+)-secoisolariciresinol diglucoside, which accumulates in the seed coat. Callus culture of *L. usitatissimum* infected by *R. rhizogenes* strain A4 developed HRs. The roots, grown in MS liquid medium, accumulated up to 4 g FW of biomass after three weeks of culture (inoculum weight: 0.5 g). In the HRs, matairesinol (MAT), secoisolariciresinol (SECO), and SECO diglucoside were detected in different proportions depending on the analyzed clone. The total content of lignans in the HRs reached 1200 μM/g DW and was higher than that in the non-transformed roots and callus of *L. usitatissimum*. SECO diglucoside and MAT were not found in the non-transformed root culture [118,119]. Markulin et al. [120] obtained HRs of *L. usitatissimum* from the leaves of two flax cultivars inoculated with *R. rhizogenes* strain 15834. A member of the WRKY transcription factor family, *Lu*WRKY36, was isolated from the ABA- or *Fusarium oxysporum*-treated cell suspension of *L. usitatissimum*. The HRs constitutively overexpressing *LuWRKY36* demonstrated increased *LuPLR1* gene transcription and a 3-fold increase in SECO accumulation (up to 6 mg/g DW). A *Fusarium*-resistant cultivar of flax exposed to the plant pathogen showed a rapid and strong increase in *LuWRKY36* expression as early as 1.5 h post elicitation, increased *LuPLR1* expression, and a significant increase in the accumulation of SECO. In the *Fusarium*-susceptible cultivar, the response was slower and weaker, and the increase in the SECO content was negligible. SECO at a concentration of 10 μM powerfully limited the growth of *F. oxysporum,* and at 100 μM, it inhibited the growth of the mycelium.

#### 2.2.4. Phrymaceae

*Phryma leptostachya* L., a medicinal plant applied as an anti-inflammatory remedy, also found use in agriculture as an insecticidal and antiviral agent. The plant synthesizes furofuran lignans such as leptostachyol acetate, 6-desmethoxy-leptostachyol acetate, (+)-phrymarolin I, and (+)-haedoxan A, some of which show insecticidal activity or act synergistically with pesticides [121]. The proposed biosynthetic pathway of the compounds includes the conversion of (+)-PINO into (+)-sesamin via (+)-piperitol. The conversion is catalyzed by the enzyme from the cytochrome P450 family, CYP81Q38. The HRs of *P. leptostachya* were initiated from the explants of aseptically grown seedlings inoculated with three different *R. rhizogenes* strains, using a culture medium supplemented with 200 μM acetosyringone. Stem segments infected with the strain ATCC 15834 were the most suitable for the induction of HRs. The HR cultures, grown for four weeks in a half MS medium, yielded 7.4–8.9 g FW/flask (0.7–0.8 g DW/flask) of the biomass, 52.17 μg/g DW 6-desmethoxy-leptostachyol acetate, and 7.27 μg/g leptostachyol acetate. The HRs with silenced *PlCYP81Q38* demonstrated significantly reduced production of 6-desmethoxy-leptostachyol acetate and leptostachyol acetate (by 73% and 86%, respectively). The genome editing CRISPR/Cas9 (clustered regularly interspaced short palindromic repeats/CRISPR-associated protein 9) technology was applied to knock out the *PlCYP81Q38* gene [122]. Stem explants of *P. leptostachya* were transformed with *R. rhizogenes* strain ATCC 15834 carrying the *PlCYP81Q38*-CRISPR/Cas9 expression vector, and as a result, four *PlCYP81Q38* knockout HR lines were obtained. The *PlCYP81Q38* mutation caused the accumulation of pinoresinol and diminished production of sesamin, 6-demethoxy-leptostachyol acetate, and leptostachyol acetate in the genetically engineered HRs. Expression of the genes encoding the enzymes of the biosynthetic pathway upstream from pinoresinol was enhanced in the *PlCYP81Q38* knockout HRs.

#### 2.2.5. Phyllanthaceae

Many species from the genus *Phyllanthus* are known for their antimicrobial, anti-inflammatory, and hepatoprotective properties [123]. The lignans phyllanthin and hypophyllanthin, synthesized by the plants, are considered as metabolites with hepatoprotective effects. Clones of the *Phyllanthus niruri* L. HRs obtained by Ishimaru et al. [124] with two strains of *Rhizobium* (*R. rhizogenes* A4 and *R. tumefaciens* R-1000 + 121) and cultivated in a half MS medium synthesized gallic acid, (−)-epicatechin, (+)-catechin, (+)-gallocatechin, (−)-epigallocatechin, (−)-epicatechin 3-O-gallate, and (−)-epigallocatechin 3-O-gallate, but lignans were not found in the tissue. In vitro-grown plants of *P. amarus* Schumach. & Thonn. inoculated with *R. rhizogenes* ATCC 15834 developed HRs. The fastest growing clone of the HRs, maintained in a one-fourth MS liquid medium supplemented with 3% sucrose under an 18/6 h (light/dark) photoperiod, yielded 9.5 g/flask of fresh biomass after four weeks of culture. A partially purified fraction of the extract from the HRs inhibited the binding reaction of HBsAg to its antibody by 86%. However, the chemical composition of the fraction was not examined [125]. Methanolic extract prepared from the HRs of *P. amarus* grown on a solidified MS medium in the dark demonstrated a concentration- and time-dependent cytotoxic effect on human adenocarcinoma (MCF-7) cells in vitro (IC_50_ ∼ 250 μg/mL at 12 h of treatment). The extract induced apoptosis in the cancer cells, but its active constituents remained unknown [126]. The HRs of *P. acuminatus* Vahl were elicited using SA (200 and 50 μM) and MJ (50 μM). The extracts from the elicited and control HRs, after chromatographic separation by UPLC (ultra-high-performance liquid chromatography), were analyzed by non-targeted high-resolution mass spectrometry. The chemical components of the extracts (including several aryltetralin lignans) were tentatively identified, based on their molecular masses and fragmentation patterns, and the heat maps were created reflecting the intensity of individual signals. Salicylic acid (50 μM) turned out to be the most effective elicitor of secondary metabolism in the *P. acuminatus* HRs. The contents of the lignans phyllanthusmin A and piscatorin increased over 2-fold after the SA treatment, but the greatest increase in content was observed for phyllamyricin D (5-fold) [127].

#### 2.2.6. Pedaliaceae

*Sesamum indicum* L., an ancient oilseed crop, provides valuable fatty oil and edible seeds. Sesame oil, due to the tocopherol, tocotrienol, and lignan contents, demonstrates antioxidative properties and is considered a health-promoting product. The major lignans present in sesame seeds are sesamin (77–930 mg/100 g), sesamolin (61–530 mg/100 g), and PINO (27–38 mg/100 g) [128]. The most noteworthy biological activities of sesame lignans are inhibitory effects on the growth of the cancer cells, estrogenic/antiestrogenic effects, neuroprotective, cardioprotective, and antiaging activity. The biosynthesis of sesame lignans, their biological activities, toxicological aspects, and biotechnological perspectives have been summarized in the comprehensive review by Andargie et al. [128].

The CRISPR/Cas9 system was used to create targeted genetic mutations in the genes encoding the enzymes converting PINO into sesamin (*CYP81Q1*) and sesamin into sesamolin (*CYP92B14*). The *R. rhizogenes* strain K599 bearing either the *CYP81Q1-gR* or *CYP92B14-gR* construct or an empty vector (control) was used to induce HRs from the cotyledons of *S. indicum*. In the obtained HRs, the mutagenesis efficiency was 90.63% for *CYP81Q1* and 93.33% for *CYP92B14*. The *CYP81Q1* knockout HR clones produce neither sesamin nor sesamolin. In *CYP92B14* knockout roots, enhanced accumulation of sesamin (up to 70 μg/g DW) and a diminished sesamolin content were observed [129]. Resequencing of 410 sesame accessions and thorough analysis of the obtained results led to the identification of 17 SNP (single nucleotide polymorphic) loci for sesamin variation, 72 SNP loci for sesamolin variation, and 11 possible causative genes. The major SNP locus for lignan variation is located in the exon of the gene *SiNST1*, encoding the transcription factor NST1, which is considered a master regulator of lignans and lignin biosynthesis in plants. Its “C” allele promotes a higher accumulation of lignans. Overexpression of *SiNST1^C^* in sesame HRs resulted in a significantly higher accumulation of sesamin and sesamolin and enhanced expression of the genes encoding enzymes of monolignol biosynthesis (*SiPAL*, *SiC4H*, *SiC3H*, *SiCCR*, *SiCOMT*, and *SiCCaAOMT*). The expression of the piperitol/sesamin synthase (PSS) encoding gene was also significantly enhanced in the *SiNST1^C^*-overexpressing HRs [130]. Dirigent proteins (DIRs) catalyze the stereoselective dimerization of coniferyl alcohol to form either (+)- or (−)-PINO. Forty-four DIR family genes were identified in *S. indicum*. They are differentially regulated by SA, MJ, and 1-aminocyclopropane-1-carboxylate. One of the genes, *SiDIR21*, was selected to prepare the construct that was introduced to *S. indicum* HRs. Roots with overexpression of *SiDIR21* produced 100.53–128.00 μg/g DW sesamin and 29.95–63.96 μg/g DW sesamolin. The contents of the lignans were up to 3.8 times higher compared to the control HRs carrying the empty vector [131].

#### 2.2.7. Taxaceae

A single study [132] was devoted to the accumulation of lignans in the HRs of *Taxus* × *media*. The HR cultures were initiated by the inoculation of in vitro-grown yew plantlets with either *R. rhizogenes* LBA 9402 (clone KT) or with *R. tumefaciens* strain C58C1 carrying the plasmid RiA4 and the plasmid pCAMBIA-TXS-His with the taxadiene synthase gene of *T. baccata* (clone ATMA). The roots were cultivated in a liquid DCR-M [133] medium in the dark and subcultured every four weeks. The ATMA HRs produced SECO (2.35 ± 0.36 μg/g DW), LARI glucoside (1.43 ± 0.31 μg/g DW), hydroxymatairesinol (HMR; 0.2 ± 0.01 μg/g DW), HMR glucosides (0.61 ± 0.05 μg/g DW), PINO (6.76 ± 2.73 μg/g DW, only in 14-day-old cultures), and PINO glucosides (12.28 ± 1.27 μg/g DW, only in 14-day-old cultures). In the KT clone of HRs, HMR (1.01 ± 0.16 μg/g DW), HMR glucosides (0.20 ± 0.01 μg/g DW), PINO glucosides (9.70 ± 1.01 μg/g DW, only in 14-day-old culture), and MAT glucosides (75.40 ± 3.15 μg/g DW) were detected. The ATMA HRs (28-day-old) were fed with coniferyl alcohol (1, 10, or 100 μM), phenylalanine (100 μM), and elicited with 100 μM MJ in different combinations and grown for another two weeks. The biomass increase in the treated HRs one and two weeks after the addition of the precursors alone or in combination with MJ was significantly lower than that of the control roots, except for the HRs concomitantly fed with 1 μM coniferyl alcohol, 100 μM phenylalanine, and elicited with 100 μM MJ. The most spectacular results were achieved when the HRs were fed with 1–10 μM coniferyl alcohol and simultaneously elicited with MJ (12.86 μg/g DW HMR after one week of the treatment; 24.29–37.88 μg/g DW HMR after two weeks). The experiment with perfluorodecalin added to the 14-day-old HR cultures to create a two-phase culture system was conducted with both KT and ATMA HRs. Elicitation of the ATMA roots grown in the two-phase (DCR-M liquid medium/perfluorodecalin aerated) culture system resulted in the production of matairesinol and PINO (159.79 ± 10.91 μg/g DW and 23.78 ± 4.92 μg/g DW, respectively, one week post elicitation; 199.86 ± 22.55 μg/g DW and 28.47 ± 8.46 μg/g DW two weeks after elicitation).

Highly productive HR cultures are summarized in Table 2.

### 2.3. Xanthones

**Figure 6:**
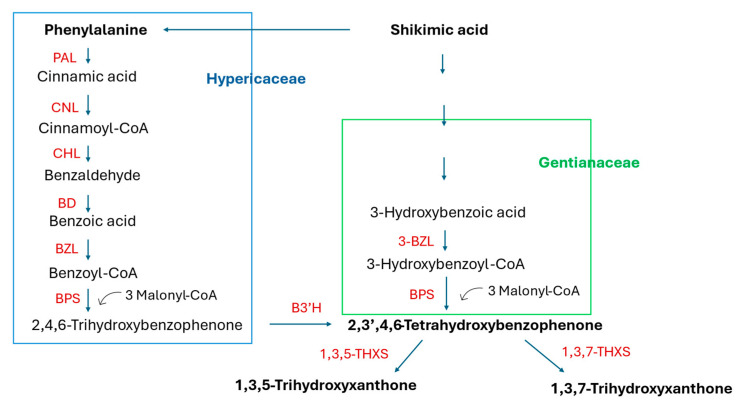
A simplified scheme of xanthone biosynthesis in plants considering different biosynthesis routes in plants of the Gentianaceae and Hypericaceae families. PAL, phenylalanine ammonia-lyase; CNL, cinnamate:CoA ligase; CHL, cinnamoyl-CoA hydratase/lyase; BD, benzaldehyde dehydrogenase; BZL, benzoate: CoA ligase; BPS, benzophenone synthase; 3-BZL, 3-hydroxybenzoate: CoA ligase; B3′H, benzophenone 3′-hydroxylase; 1,3,5-THXS, 1,3,5-trihydroxyxanthone synthase; 1,3,7-THXS, 1,3,7-trihydroxyxanthone synthase.

Xanthones are a class of polyphenolic compounds characterized by a dibenzo-γ-pyrone scaffold, consisting of two benzene rings (A and B) connected via a central pyranone ring (C6–C1–C6 skeleton) [134]. This unique tricyclic structure allows for extensive substitution patterns, giving rise to a wide variety of natural derivatives with diverse biological functions. Naturally occurring xanthones are mainly found in higher plants, particularly in the families Iridaceae, Moraceae, Gentianaceae, Clusiaceae, and Hypericaceae, as well as in fungi, lichens, and marine microorganisms [134,135,136]. In plants, xanthones are synthesized via two different biosynthetic pathways (see Figure 6). Numerous studies have highlighted the broad pharmacological potential of xanthones, including antioxidant [20,137], anti-inflammatory [20,137,138], anticancer [20,21,139,140,141,142], antimicrobial [20,137,143], antihyperglycemic [144,145], and cardio- and neuroprotective activities [20,146,147]. Mangiferin, one of the most studied xanthones, demonstrated promising effects in diabetes remediation and neurodegenerative disease prevention and as a cardio-, hepato-, gastro-, and nephroprotective agent [148,149]. Due to their structural diversity and bioactivity, xanthones are being actively explored in the context of phytopharmaceuticals, nutraceuticals, and cosmeceuticals. In vitro culture systems, such as hairy root cultures, are increasingly used to study xanthone biosynthesis under controlled conditions.

#### 2.3.1. Fabaceae

**Figure 7:**
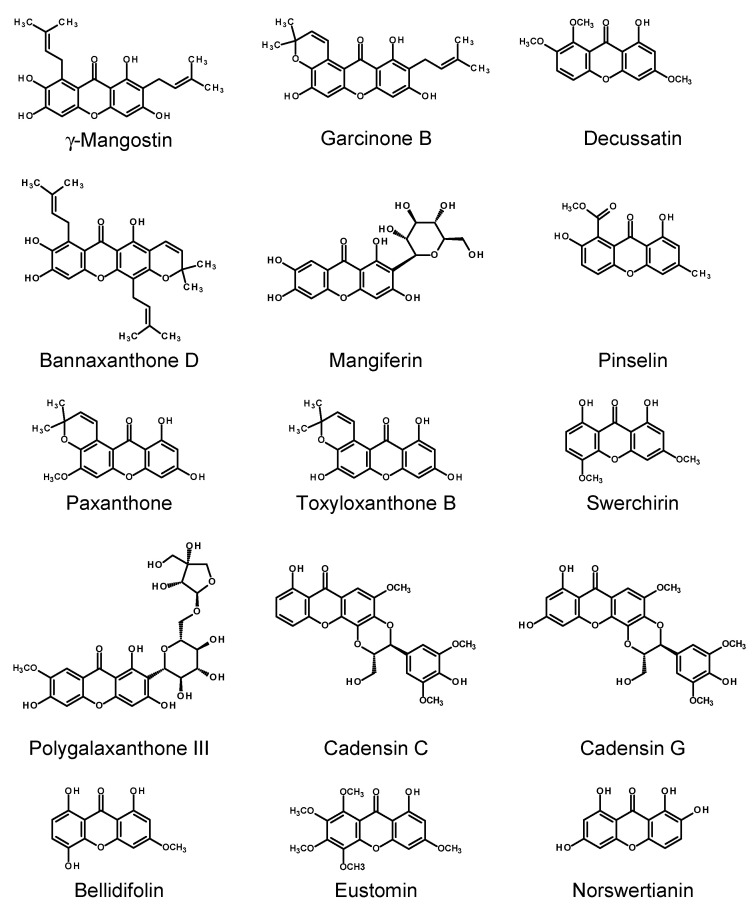
Chemical structures of selected xanthones produced by the HRs of *Cassia occidentalis* (pinselin), *Centaurium* spp. (eustomin, decussatin), *Gentiana* spp. (norswertianin), *Swertia* spp. (bellidifolin, swerchirin), *Hypericum* spp. (mangiferin, paxanthone, γ-mangostin, cadensins C and G, garcinone B, bannaxanthone D, toxyloxanthone B), and *Polygala tenuifolia* (polygalaxanthone III).

*Cassia occidentalis* L. (= *Senna occidentalis* (L.) Link) is an antraquinone-containing plant native to America. HRs of *C. occidentalis*, induced with two *R. rhizogenes* strains, LBA 9402 and A4, produced the xanthone pinselin (39–145 μg/g FW; for the structure, see Figure 7). Nitsch and Nitsch nutrient medium supplemented with 3% sucrose was optimum for the accumulation of pinselin and the antraquinone germichrysone in the transformed roots [150].

#### 2.3.2. Gentianaceae

*Swertia japonica* (Schult.) Makino has been used in Japan as a bitter stomachic called ‘senburi’ and has been claimed to be effective in hepatitis. HRs of *S. japonica*, derived from the axenic shoots inoculated with *R. rhizogenes* ATCC 15834, produced four xanthones: 8-*O*-primverosylbellidifolin, bellidifolin (see Figure 7), methylbellidifolin (swerchirin), and swertianolin. Based on the isolation yields, the contents of individual xanthones in the HRs were no less than 0.2–1.9 mg/g DW [151]. The HRs of *S. chirayita* (Roxb.) H.Karst. were induced via inoculation of the leaves of aseptic plantlets with *R. rhizogenes* strain LBA 9402. The roots, cultivated in a liquid MS medium, accumulated 13.32 ± 0.50 g/flask FW and 1.29 ± 0.05 g/flask DW of biomass in a 60-day growth cycle. The HR growth could be improved by the application of a half MS medium with a diminished content of sucrose (2%). Two xanthones, swerchirin and formerly undescribed 1,2,5,6-tetrahydroxyxanthone, were isolated from the HRs and identified based on their spectral data. At the stationary phase of the culture, the contents of swerchirin and 1,2,5,6-tetrahydroxyxanthone in the HRs reached 0.39 and 0.91 mg/g DW, respectively. Attempts were made to stimulate the xanthone biosynthesis by elicitation. The HRs were elicited by the addition of MJ (10, 25, 50, and 100 μM), SA (10, 25, 50, and 100 μM), or sodium nitroprusside (5, 10, 20, and 40 μM) to the 60-day-old cultures. The contents of xanthones in the elicited HRs were assessed after three and six days of treatment. The best production of 1,2,5,6-tetrahydroxyxanthone was achieved using 100 μM MJ (5.501 ± 0.73 mg/g DW), 50 μM SA (4.411 mg/g DW), or 10 μM sodium nitroprusside (4.126 mg/g DW), six days post elicitation. The content of swerchirin in the elicited HRs did not differ significantly from that in the control roots [152,153].

Quantitative analysis of the xanthone content in HR cultures of *Centaurium pulchellum* (Sw.) Druce and *C. erythraea* Rafn. transformed with *R. rhizogenes* strain A4M70GUS revealed notable interspecies and clone-specific differences. In *C. pulchellum*, the HR clone G accumulated 1.50 ± 0.03% DW methylbellidifolin, while demethyleustomin was the dominant xanthone produced by clone H (3.26 ± 0.04% DW). Decussatin was a minor compound in the examined *C. pulchellum* HRs. The HRs of *C. erythraea* accumulated up to 1.21 ± 0.04% DW eustomin and up to 0.43% DW demethyleustomin. There was no obvious difference in the productivity between the dark-grown roots and the roots cultivated under the photoperiod. The HR cultures were maintained on a solidified MS medium, and spontaneous regeneration of shoots from the HRs was observed [154].

An HPLC analysis of methanolic extracts from *Gentiana dinarica* Beck HRs, induced with the agropine *R. rhizogenes* strains A4M70GUS and 15834/PI, confirmed the presence of the key xanthones norswertianin-1-*O*-primeveroside, norswertianin-1-*O*-glucoside, gentioside, and norswertianin in all tested clones. The xanthone accumulation varied with both carbohydrate type and its concentration in the nutrient medium. Norswertianin-1-*O*-primeveroside was the dominant xanthone across all clones, with the highest accumulation (32.4 mg/g DW) observed in clone 3, grown in the half MS medium with 4% sucrose. Similar trends were seen for the norswertianin-1-*O*-glucoside and gentioside contents, which also peaked at this sucrose concentration. Glucose and fructose supported maximum xanthone production at 175.2 mM (3.2%) concentration. In contrast, the aglycone norswertianin showed decreased accumulation with rising sugar concentrations, accumulating primarily at lower sugar levels, particularly in thin, fast-growing roots. The medium containing 4% sucrose was optimum for the biomass accumulation (over 500 mg DW/flask) [155]. A three-day treatment with abiotic elicitors (SA, MJ, jasmonic acid) significantly stimulated the production of the main xanthone norswertianin-1-*O*-primeveroside in clone 3 of *G. dinarica* HRs (obtained with *R. rhizogenes* strain 15834/PI) but did not affect the productivity of clone D (obtained with A4M70GUS). A longer seven-day treatment generally reduced the levels of norswertianin-1-*O*-primeveroside. In contrast, norswertianin aglycone was significantly upregulated in both HR clones following seven-day treatments with SA (200 μM) or jasmonic acid (200 μM). In clone 3 of HRs, MJ (50, 100, and 200 μM) also significantly increased the aglycone content. Yeast extract had little effect on norswertianin-1-*O*-primeveroside accumulation, while chitosan enhanced its production in clone 3 at concentrations from 5 to 20 mg/L. The norswertianin contents increased markedly with higher concentrations of both YE (5 g/L) and chitosan (50 mg/L). In clone 3, YE and chitosan induced 8- and 24-fold increases, respectively, while clone D exhibited 16- and 15-fold enhancements under the same treatments. The biotic elicitors (YE and chitosan) stimulated the growth of the HRs, except for the highest dose of chitosan (50 mg/L). New xanthones (absent from the controls) such as isogentisin and another unidentified xanthone, were detected in the HRs after the chitosan and YE treatments [156]. It is worth noting that xanthone-rich extract from the HRs of *G. dinarica* and norswertianin alone induced autophagy in a human glioblastoma cell line in vitro [157]. Vinterhalter et al. [158] examined four distinct clones of *G. dinarica* HRs, obtained as described in the earlier studies of the same research team [155,156]. The clones (cl-B, cl-D, cl-3, and cl-14) were maintained in a hormone-free MS medium. A comparative cultivation of the HRs in Erlenmeyer flasks, temporary immersion systems (TISs RITA^®^; Vitropic, St-Mathieu de Tréviers, France), and bubble column bioreactors (BCBs) revealed that the aerated bioreactors (TISs and BCBs) significantly enhanced both biomass accumulation and xanthone production relative to the culture in flasks, highlighting the importance of oxygen availability in metabolite biosynthesis. Clone cl-B consistently outperformed the others, achieving the highest growth index and dry biomass in BCBs. Nonetheless, superior accumulation of norswertianin-1-*O*-primeveroside took place in the temporary immersion conditions (TISs). Clone cl-B was also characterized by the highest norswertianin accumulation in BCBs at 2% sucrose concentration. Notably, the sucrose concentration exerted differential effects: increasing sucrose from 2% to 4% boosted norswertianin-1-*O*-primeveroside production by up to 50%, whereas the norswertianin content was markedly higher at 2% sucrose, especially in BCB-grown HRs of clones cl-B and cl-3, suggesting distinct sugar-dependent regulatory mechanisms for these metabolites [158].

*G. utriculosa* L., HR line 9 was obtained by transformation with *R. rhizogenes* strain A4M70GUS. During cultivation of the explants infected with the bacterium, apart from the HRs, callus tissue emerged, and somatic embryos developed spontaneously. Plants regenerated from the embryos were genetically transformed, and their DNA contained fragments of the bacterial T-DNA. Line 9 of HRs demonstrated the best growth on a solid half MS medium (GI = 1.4 after 35 days of growth) and at the same time showed the highest regeneration potential and the highest number of somatic embryos per culture. The root line produced 23.74 mg/g DW of decussatin-1-*O*-primeveroside, much more than the roots collected from nature (10.26–10.35 mg/g DW) and more than the in vitro-grown adventitious roots (18.23 mg/g DW) and regenerated shoots. Moreover, decussatin and smaller amounts of other xanthones (mangiferin, gentiakochianin 1-*O*-primeveroside, 1,8-dihydroxy-3-methoxyxanthone 7-*O*-primeveroside, gentiakochianin, gentiacaulein) were detected in the roots [159].

#### 2.3.3. Hypericaceae

*Hypericum perforatum* L., is the most well-known and the most intensively studied species of the Hypericaceae. The methods of elicitation to improve the profiles of high-value secondary metabolites in the plant and its tissue cultures, genetic engineering prospects for this species, and advances in tissue culture of *H. perforatum* have already been summarized by several reviews [160,161,162]. To the best of our knowledge, the first HR cultures of *H. perforatum* were obtained in the early 2000s [163], but the first study devoted to xanthones produced by the HRs was published by Tusevsky et al. [164]. The HRs of *H. perforatum* were initiated from the root explants inoculated with *R. rhizogenes* strain A4 and cultivated on a solidified MS/GB5 medium in the dark. The metabolic profile of the HRs was analyzed using the HPLC-DAD-ESI-MS^n^ technique. The obtained root clones demonstrated significantly enhanced xanthone accumulation compared to non-transformed roots, with several xanthones synthesized de novo post-transformation (1,3,6,7-tetrahydroxyxanthone, 1,3,6,7-tetraxydroxy-4-(1,1-dimethylallyl)-xanthone, γ-mangostin, garcinone C, paxanthone B, 1,3,7-trihydroxy-2-(2-hydroxy-3-methyl-3-butenyl)-xanthone, 1,3,5,6-tetrahydroxyxanthone) [164,165]. In the following study, the *H. perforatum* HRs were analyzed for phenolic compound production under the dark and photoperiod conditions. The roots grown under the photoperiod showed increased levels of seven xanthones, along with the de novo synthesis of bannaxanthone E, tetrahydroxy-1-methoxyxanthone, 1,3,5-trihydroxy-6-methoxyxanthone, paxanthone, and 1,3,5,6-tetrahydroxy-2-prenylxanthone [166]. Follow-up research by Tusevski et al. [167] reaffirmed the effect of light conditions on metabolite production in *H. perforatum* HRs. The HRs grown in the dark produced higher amounts of vanillic acid, syringic acid, flavan-3-ols, and flavonol glucosides, whereas the HRs grown under the photoperiod accumulated elevated levels of naphthodianthrones (pseudohypericin and protopseudohypericin) and xanthones [167]. However, these results should be interpreted with caution, as the data for the HRs under the photoperiod and in the dark conditions overlap in two publications [164,166]. Transgenic shoots, transformed with *R. rhizogenes* strain A4, exhibited distinct profiles of xanthone derivatives, including both identified compounds (e.g., mangiferin, garcinone C, banaxanthone E) and a series of putative, unidentified xanthone derivatives (designated as X3, X4, X5, etc.) [165]. 1,3,6,7-Tetrahydroxyxanthone dimer and 1,3,6,7-tetrahydroxyxanthone were found exclusively in the non-transformed shoots, while paxanthone B was synthesized only in the transformed shoots. Trihydroxy-1-methoxy-C-prenyl xanthone (6.23–22.8 mg/g DW), mangiferin (0.03–8.40 mg/g DW), and the glycosidic form of 1,6-dihydroxy-3,5-dimethoxy xanthone were the major xanthones found in the HRs grown in a liquid medium (GI: 7.75–12.37, calculated on the FW basis) [165]. In earlier research [164,166], it was shown that the HRs grown on a solidified medium, in the dark or under the photoperiod, accumulated mainly trihydroxy-1-methoxy-C-prenyl xanthone (100.67–113.14 mg/g DW) and garcinone E (82.29–108.44 mg/g DW). In 2023, Tusevski and coworkers re-examined the HRs of *H. perforatum* grown in Petri dishes on a solidified medium [168]. Except for the HPLC-MS analysis, the effects of the extracts prepared from the HRs on the activities of selected enzymes (monoaminoxidase-A, acetylocholinesterase, tyrosinase, α-amylase, and α-glucosidase) were investigated. A total of 38 xanthones were identified in both transgenic HR cultures and untransformed roots of *H. perforatum*. Several xanthones were unique to untransformed roots. The compounds were grouped by concentration: the first group (0.3–0.8 mg/g DW) included trihydroxy-1-methoxy-C-prenyl xanthone and 1,3,6-trihydroxy-7-methoxy-8-prenyl xanthone, while the second group (≤0.3 mg/g DW) comprised 1,3,6,7-tetrahydroxyxanthone dimer, 1,3,7-trihydroxy-6-methoxy-8-prenyl xanthone, and 5-O-methylcelebixanthone. In the HR cultures, two compounds were synthesized de novo in all clones. Six xanthones, homomangiferin, 3,6-dihydroxy-1,5,7-trimethoxy-xanthone, 5-O-methyl-2 deprenylrheediaxanthone B, and cadensin G, were specific to the clone HR B. Linixanthone C and isomangiferin were exclusive to HR H, while γ-mangostin isomer appeared only in HR F. Among all lines, HR B exhibited the highest total xanthone content, highlighting its potential as a high-yielding producer [168].

Brasili et al. [169] demonstrated that elicitation with chitosan enhanced brasilixanthone B accumulation in *H. perforatum* in vitro root cultures. The effects of SA (50, 100, and 250 μM) and jasmonic acid (10, 50, and 100 μM) on phenolics production in the HRs of *H. perforatum* were investigated by Gjureci et al. [170]. The HRs grown in a liquid nutrient medium containing MS salts and GB5 vitamins in the dark were treated with elicitors 7 days after inoculation in fresh medium. SA, in the applied concentration range, significantly reduced the growth of the HRs (with 100 and 250 μM SA, the growth was severely limited), while only the highest concentration of jasmonic acid exerted negative effects on the growth of the roots. The HPLC profiling identified 23 xanthones in both control and elicited HR cultures. Some xanthones were exclusive to control roots, while elicitor treatments induced de novo biosynthesis of others. The elicitation with SA triggered the synthesis of two new xanthones and markedly increased the levels (2.7- to 10-fold) of compounds such as tetrahydroxyxanthone-C-pentoside and 1,3,5,6-tetrahydroxyxanthone. Jasmonic acid primarily upregulated a distinct group of prenylated xanthones, including α-mangostin and garcinone E. The total xanthone content increased more than 2-fold with jasmonic acid treatment, whereas SA elicitation led to comparatively lower overall xanthone accumulation [170].

According to Zubrická et al. [171], who investigated the xanthone accumulation in adventitious roots, HRs, and cell suspension cultures of several *Hypericum* species, the highest xanthone contents were found in untransformed roots of *H. pulchrum* L. and *H. annulatum* Moris. Comparative metabolic profiling of different *Hypericum* species grown under various light treatments revealed notable interspecific variation in the xanthone accumulation. While growing in the dark, the roots of *H. pulchrum* and *H. annulatum* exhibited the highest xanthone accumulation (2.74–4.88 mg/g DW), with three compounds common to both species, paxanthone unique to *H. pulchrum*, and methoxy-2-deprenylrheediaxanthone B exclusive to *H. annulatum*. Even though the roots of *H. pulchrum* had the greatest xanthone content, they demonstrated the slowest growth rate. The other slowly growing roots (*H. humifusum* L. and *H. kouytchense* H.Lév.) showed minimal xanthone levels. Under a 16/8 h (light/dark) photoperiod, xanthone concentrations ranged from 0.47 to 1.37 mg/g DW, with *H. tomentosum* L. presenting the highest content despite the reduced growth. Mangiferin was detected across all species, whereas methoxy-2-deprenylrheediaxanthone B was specific to *H. tetrapterum* Fr. Key xanthones (1,3,6,7- and 1,3,5,6-tetrahydroxyxanthone) were present in all species but *H. rumeliacum* Boiss., with *H. tomentosum* showing 2- to 3-fold higher levels compared to *H. tetrapterum*, *H. maculatum*, and *H. annulatum*. In the HRs, the total xanthone contents varied from 0.11 to 2.02 mg/g DW. One of the transgenic *H. tetrapterum* root lines exhibited approximately a 30-fold increase in the 1,7-dihydroxyxanthone content (0.16 mg/g DW) relative to the control (0.005 mg/g DW). The *H. tomentosum* HRs contained toxyloxanthone B and both tetrahydroxyxanthones, although the total xanthone content increased less than 2-fold compared to the control roots (0.97 mg/g DW). The xanthone composition varied notably between hairy root clones and their respective untransformed roots under both photoperiod and darkness conditions [171].

#### 2.3.4. Polygalaceae

The highest transformation efficiency (85.83%) in *Polygala tenuifolia* Willd. was achieved using *R. rhizogenes* strain C58C1 co-cultured for 3 days on a solidified half MS medium supplemented with 200 μM acetosyringone. The obtained HR cultures were optimized for biomass and metabolite production using four nutrient media, namely, MS, GB5, N6 [172], and 6.7-V [173], and induction with brassinolide and trehalose. Starting with 0.5 g FW inoculum, 3.49 g/flask FW and 0.41 g/flask DW were produced after 6 weeks of culture in the 6.7-V medium supplemented with 3% sucrose. Polygalaxanthone III (5.55 mg/g DW) (for structure, see Figure 7) was the major secondary metabolite synthesized by the HRs. The accumulation of polygalaxanthone III was enhanced by changing the carbon source from sucrose to either a mixture of glucose and sucrose (c. 12 mg/g DW after four weeks) or to fructose (c. 8 mg/g DW after six weeks). The induction of the 5-week-old HRs with 15 μM trehalose led to the stimulation of growth (5.64 g/flask FW and 0.54 g/flask DW), but the highest content of polygalaxanthone III (13.38 mg/g DW) was found in the HRs induced with the 10 μM concentration. Brassinolide (1 × 10^−12^ M) enhanced the biomass accumulation (6.31 g/flask FW and 0.52 g/flask DW), but maximum polygalaxanthone III production (over 14 mg/g FW) was achieved with 1 × 10^−8^ M of the plant hormone. The estimated content of polygalaxanthone III in the HRs was much higher than that usually found in roots of the parent plant [174].

The HRs that demonstrated reasonable growth rates and accumulated high contents of xanthones are listed in Table 3.

### 2.4. Coumarins, Lignans, and Xanthones: Variety of Sources and Prospects for Their Production by HRs

Some of the coumarins, lignans, and xanthones synthesized and accumulated by plants are simple chemical scaffolds that are easily accessible by a chemical synthesis. Numerous processes leading to these natural products have been developed and described in the literature [175,176,177,178]. However, for economic reasons, only some of them can be used for synthesis on a larger scale. To reduce the environmental costs of the chemical synthesis, “green technologies” such as catalytical synthesis and microwave-assisted synthesis are increasingly the subject of interest of organic chemists.

Plants are not the only source of biologically active coumarins, lignans, and xanthones [85,134,135,179]. Cultures of microorganisms, bacteria, and fungi of both marine and terrestrial origin can provide target compounds with many potential applications or furnish substrates for the semi-synthesis of the target active compounds. The enzymatic potential of microorganisms has been utilized to transform coumarin into its less toxic derivative dihydrocoumarin [180,181]. Genetically modified microorganisms (GMMs) are currently perceived as those biological systems capable of biosynthesizing active molecules that are relatively easier to scale up for production purposes than in vitro plant cultures. However, they also have their limitations. Microbial cell factories are often susceptible to the cytotoxic effects of the target products and usually need externally supplied substrates to initiate and conduct processes [182]. Genetically engineered strains of *Escherichia coli* produced scopoletin, esculetin, and umbelliferone. In a fed-batch experiment, the production of esculetin with a high titer (855.5 mg/L within 120 h) has been achieved [183]. The budding yeast (*Saccharomyces cerevisiae*) was engineered to convert lignin monomers to scopoletin and fraxetin. In the lignin valorization process, a lignin hydrolysate was converted to scopoletin with a titer of 4.79 ± 0.72 mg/L (96 h) [184]. The complete biosynthesis of osthole, a prenylated coumarin, has been achieved in engineered yeast, and the titer of the compound reached 108.10 mg/L in shake flask cultures and 255.1 mg/L in fed-batch fermentation (120 h) [185]. Ten products of the lignan biosynthetic pathway in *I. indigotica*, namely, (+)-lariciresinol, (−)-secoisolariciresinol, (−)-matairesinol, and their glycosides, were synthesized by genetically engineered *E. coli* using eugenol as a substrate [186]. This would not have been possible without previous biosynthesis studies using HR cultures [79,80,81,82,83]. Recently, de novo synthesis of the key lignan molecules pinoresinol and lariciresinol has been achieved in a synthetic yeast consortium [187].

Except for the biosynthesis in genetically engineered microorganisms, heterologous synthesis of target metabolites in genetically modified plants of readily accessible species has also been considered. Heterologous production of sesame lignans has been achieved in transgenic *Forsythia* plants, and *Nicotiana benthamiana* seems to be a promising heterologous host to produce podophyllotoxin-related lignans [188].

However, in many cases, plants remain the raw material for the extraction of biologically active compounds. Traditional sources of coumarin are tonka bean (*Dipteryx odorata* Wild; from 20.4 ± 0.4 to 43.4 ± 0.9 mg/g of coumarin) and barks of various species of *Cassia* and *Cinnamomum* (e.g., *Cinnamomum loureiroi* Nees; 1.06 g/kg of coumarin) [189,190]. Plants from the Apiaceae family are known as sources of furanocoumarins, pyranocoumarins, and prenylated coumarins. Furanocoumarins accumulate mainly in the seeds of these plants and may be why the HRs of some Apiaceae plants do not meet expectations in terms of coumarin production efficiency. Dried roots of *Angelica gigas* Nakai contain pyranocoumarins, decursin (2.5–5%, w/w), and decursinol angelate (2–3.5%) [191]. The content of decursinol angelate is much higher than that in the genetically engineered HRs of *A. gigas* [41]. *Ferula* spp. are especially rich in coumarins of diverse structures, but little is known about the biotechnological production of these compounds. Recently, a review on the possible limitations of secondary metabolite production in *Ferula* spp. by biotechnological techniques has been published that highlights the possible role of endophytes in metabolite biosynthesis [192]. The content of scopoletin in the roots of selected plant species ranges from 0.0001% in *Angelica pubescens* Maxim. to 0.01125% in *Brunfelsia hopeana* Benth., but the contents of the compound in the aerial parts of Rutaceae and Burseraceae plants are much higher (up to 0.98%) [193].

The richest sources of plant lignans are knots of Norway spruce (*Picea abies* (L.) H. Karst) that contain 6–24% lignans, with 7-hydroxymatairesinol as the major compound [194]. The best dietary sources of lignans (from 132 to 835 mg/100 g) are sesame and flax seeds [70,71,195]. The content of podophyllotoxin in roots and rhizomes of *Podophyllum hexandrum* Royle from the Indian Himalayas ranged from 0.012 to 5.480% DW, and similar or higher contents (up to 8% DW) of podophyllotoxin-related lignans were found in the HRs of *Linum* spp. [92,93,96,100,103,109,115,116,117,196].

Pericarps of mangosteen (*Garcinia mangostana* L.) are the most popular source of the plant xanthones α-mangostin and γ-mangostin. The pericarp constitutes 83% of the mangosteen fruit weight. A microwave extraction of the dry pericarp allows for obtaining α-mangostin with a yield of up to 120.68 mg/g, although the yields usually range from 28 to 57 mg/g DW [197,198].

Genetic engineering techniques have so far been applied to a limited extent to increase the efficiency of coumarin, lignan, and xanthone production in HRs. Overexpression of the AgPAL and AgC4H genes was achieved in *A. gigas* HRs [41], but the engineered roots produced much less decursinol angelate than the roots of the parent plant. On the other hand, HRs of *M. albus* overexpressing *MaCYP82L1* were found to contain two–three times more coumarin than the control roots [64]. The techniques of molecular biology and genetic engineering have been applied to the widest extent in the research on the biosynthesis of lignans in *I. indigotica* [78,79,80,81,82,83,84]. Except for the overexpression of the genes encoding individual enzymes responsible for the biosynthesis of lignans, gene silencing techniques such as RNA interference (RNAi) and CRISPR/Cas9-generated knockout were applied to elucidate the lignan biosynthetic pathway and its regulation. The studies, however, were not focused on achieving HR cultures with maximum performance of target products. The silencing of PINO/LAR reductase in the HRs of *L. perenne* [100] and L. album [107], as well as the overexpression of LuWRKY36 in L. usitatissimum HRs [120], knockout of *PlCYP81Q38* in *P. leptostachya* HRs [122], and knockout of *CYP81Q1* and *CYP92B14* in sesame HRs [129] were also performed to study biosynthetic pathways. The only attempts at genetic manipulations aimed at maximizing lignan production in HRs were to obtain overexpression of the transcription factor SiNST1^C^ and overexpression of the dirigent protein SiDIR21 in the HRs of *S. indicum* [130,131]. The HR cultures of *L. lewisii* [93], *L. austriacum* [96], and *G. dinarica* [158] have been successfully scaled up from Erlenmeyer flasks to bioreactors.

The prospects of HR cultures as the producers of biologically active natural products, including the application of genetic engineering methods and large-scale cultivation in bioreactors, have been extensively discussed in the recent review by Mirmazloum et al. [8]. HRs obtained with the wild strains of *R. rhizogenes* (without any genetic constructs) and plants regenerated from them are not subject to GMO regulations [199,200,201]. In fact, traces of previous transformation with *Rhizobium* can be found in the genome of healthy plants [202]. So, the companies producing GMO-free products can use biochemicals synthesized in the wild-type HRs.

## 3. Conclusions

The last 30 years have brought great progress in the research on plant in vitro cultures, including HR cultures. In addition to the old methods of stimulating the productivity of cultures (manipulations with mineral salt composition or the carbon source in the nutrient medium, the use of biotic and abiotic elicitors, supplementation with vitamins and PGRs), new ones have appeared (elicitors with new structures and more potent activity, new methods in molecular biology and genetic engineering). This has allowed for greater insight into the biosynthesis of biologically active compounds. Transformed roots (HRs) have played an invaluable role in these studies. There are many alternative ways to obtain coumarins, lignans, and xanthones, and only those that are economically effective will find practical application. Coumarins seem to be difficult to obtain from HRs with reasonable yields, though some structural types of the compounds may have some potential (sesquiterpene coumarin ethers). On the other hand, HRs are effective producers of some kinds of lignans. Attempts to scale up HR cultures using various kinds of bioreactors are noteworthy. The production of xanthones in HRs seems to be a relatively poorly studied research area.

Nutrient media containing higher concentrations of sucrose often stimulates growth of the HRs and the accumulation of glycosylated forms of the products. Elicitation procedures sometimes trigger hydrolysis of the glycoslated forms of active compounds, not de novo synthesis of the active aglycones. In experimental studies, the balance between the pool of free and glycosylated forms of the compounds of interest should be carefully monitored.

## Figures and Tables

**Table 1:** Hairy root cultures that produced high yields of coumarins: type of culture, target products, and their potential applications.

Plant Species	Hairy Roots	Natural Product	Potential Application	Maximum Content in the Biomass	Literature
*Ammi majus* L.	Wild type	Umbelliferone	Fluorescent probe, sunscreen agent	32 μg/g DW	[43]
*Angelica gigas* Nakai	Expressing *AgC4H*	Decursinol angelate	Anti-inflammatory, neuroprotective, and antiproliferative agent	0.41 mg/g DW	[41]
*Artemisia annua* L.	Undefined *R. rhizogenes* strain	Drimartol A	MDR-reversing agent	2 mg/g DW	[48]
*Cichorium intybus* L.	Wild type elicited with *Phytopthora parasitica* var. *nicotiana*	Esculin Esculetin	Treatment of varicose veins	100 μg/g FW 68 μg/g FW	[51]
*Ruta graveolens* L.	Wild type	Isopimpinelin Bergapten	PUVA therapy	1060 ± 340 μg/g DW 1010 ± 360 μg/g DW	[32]
*Sphaeralcea angustifolia* (Cav) G. Don	ATCC 15834/pTDT	Scopoletin	Fluorescent probe, antifungal, anti-inflammatory, antiarthritic, and anticancer agent	0.011 ± 0.002 mg/g DW	[59]
*Urena lobata* L.	Wild type	Imperatorin	PUVA therapy, anti-inflammatory agent	0.14 μg/g DW	[60]
*Pelargonium sidoides* DC	Wild type elicited with 100 μM MJ	Umckalin	Treatment of respiratory diseases	427.37 μg/g DW	[67]

**Table 2:** Hairy root cultures that produced high yields of tetrahydrofuran and aryltetralin lignans: type of culture, target products, and their potential applications.

Plant Species	Hairy Roots	Natural Product	Potential Application	Maximum Content in the Biomass	Literature
*Leontopodium nivale* ssp. *alpinum* (Cass.) Greuter	Wild type 6% sucrose	Leoligin 5-Methoxy-leoligin	Antihypercholesterolemic agents	0.068% DW 0.037% DW	[72]
*Isatis indigotica* Fortune	Overexpressing *IiPLR1*	Lariciresinol	Hypoglycemic, antiviral, allelopathic, and antifungal agent	353.9 μg/g DW	[78]
*Isatis indigotica* Fortune	Overexpressing *IiC3H*	Lariciresinol	Hypoglycemic, antiviral, allelopathic, and antifungal agent	96.4 mg/g DW	[80]
*Linum flavum* L.	Wild type	6-MPTOX	Anticancer agent	35 mg/g DW	[89]
*Linum tauricum* Willd.	Wild type, elicited: 150 μM MJ	4′-Demethyl-6-MPTOX 6-MPTOX	Anticancer agent	31.9 mg/g DW 36.2 mg/g DW	[92]
*Linum lewisii* Pursh	Wild type, elicited: 1 μM coronatin	Justicidin B	Anticancer agent	Over 40 mg/g DW	[93]
*Linum austriacum* L.	Wild type Stirred tank bioreactor	Justicidin B	Anticancer agent	21 mg/g DW	[96]
*Linum perenne* Himmelszelt	Wild type	Justicidin B	Anticancer agent	37 mg/g DW	[100]
*Linum mucronatum* Bertol ssp. *mucronatum*	Wild type (mikomopine strain A13)	6-MPTOX PTOX	Anticancer agent	41.4 mg/g DW 5.6 mg/g DW	[103]
*Linum album* Kotschy ex Boiss.	Wild type	6-MPTOX	Anticancer agent	48 mg/g DW	[109]
*Linum album* Kotschy ex Boiss.	Wild type, elicited: chitosan 200 mg/L	6-MPTOX	Anticancer agent	39 mg/g DW	[115]
*Linum album* Kotschy ex Boiss.	Wild type putrescine 0.25 mM	6-MPTOX	Anticancer agent	80 mg/g DW	[116]
*Linum flavum* L.	Wild type 6% sucrose	6-MPTOX free and glucosylated	Anticancer agent	65 mg/g DW	[117]

**Table 3:** Hairy root cultures that produced high yields of xanthones: type of culture, target products, and their potential applications.

Plant Species	Hairy Roots	Natural Product	Potential Application	Maximum Content in the Biomass	Literature
*Swertia chirayita* (Roxb.) H. Karst.	Wild type, elicited: 100 μM MJ	1,2,5,6-Tetrahydroxyxanthone Swerchirin	Swerchirin as a hypoglycemic agent	5.50 mg/g DW 0.40 mg/g DW	[153]
*Centaurium pulchellum* (Sw.) Druce	Wild type	Methylbellidifolin (Swerchirin) Demethyleustomin	Hypoglycemic agent Antimutagenic agent	15.0 mg/g DW 32.6 mg/g DW	[154]
*Centaurium erythraea* Rafn	Wild type	Eustomin Demethyleustomin	Antimutagenic agent	12.1 mg/g DW 0.43 mg/g DW	[154]
*Gentiana dinarica* Beck	Wild type 6% sucrose	Norswertianin 1-O-primeveroside Neoswertianin-1-O-glucoside	Aglycone as anti-glioma agent	32.4 mg/g DW 6.00 mg/g DW	[155]
*Hypericum perforatum* L.	Wild type	Trihydroxy-1-methoxy-C-prenyl xanthone Mangiferin	Mangiferin as an antioxidant, neuroprotective, hypoglycemic, and anti-inflammatory agent	6.23–22.8 mg/g DW 0.03–8.40 mg/g DW	[165]
*Polygala tenuifolia* Willd.	Wild type, elicited: 10 μM trehalose	Polygalaxanthone III	Anti-inflammatory and hypoglycemic agent	13.38 mg/g DW	[174]
*Polygala tenuifolia* Willd.	Wild type Brassinolide 1 × 10^−8^ M	Polygalaxanthone III	Anti-inflammatory and hypoglycemic agent	Over 14 mg/g DW	[174]

## Data Availability

No new data were created or analyzed in this study. Data sharing is not applicable to this article.

## Abbreviations

1:3,5-THXS	1,3,5-Trihydroxyxanthone synthase
1,3,7-THXS	1,3,7-Trihydroxyxanthone synthase
3-BZL	3-Hydroxybenzoate:CoA ligase
4CL	4-Coumaroyl:CoA-ligase
6-MPTOX	6-Methoxypodophyllotoxin
B3′H	Benzophenone 3′-hydroxylase
B5	Gamborg’s B5 medium
BD	Benzaldehyde dehydrogenase
BPS	Benzophenone synthase
BZL	Benzoate:CoA ligase
C2H	Cinnamate 2-hydroxylase
C3H	Coumarate 3-hydroxylase
C4H	Cinnamate 4-hydroxylase
CAD	Cinnamyl alcohol dehydrogenase
CaMV	Cauliflower mosaic virus
CCoAOMT	Caffeoyl-CoA O-methyl transferase
CCR	Cinnamoyl-CoA reductase
CHL	Cinnamoyl-CoA hydratase/lyase
CNL	Cinnamate:CoA ligase
COMT	Caffeic acid O-methyl transferase
CRISPR/Cas9	Clustered regularly interspaced short palindromic repeats/
	CRISPR-associated protein 9
DAD	Diode array detector
DIR	Dirigent protein
DW	Dry weight
ESI-MS	Mass spectrometry with electrospray ionization
F5H	Ferulate 5-hydroxylase
F6H	Feruloyl-CoA 6-hydroxylase
FW	Fresh weight
GA_3_	Giberelic acid
GC-MS	Gas chromatography with mass spectroscopic detection
GI	Growth index
GPX	Glutathione peroxidase
GUS	*β*-Glucuronidase
HMR	Hydroxymatairesinol
HPLC	High-performance liquid chromatography
HR	Hairy root
LARI	Lariciresinol
MAT	Matairesinol
MDA	Malonyl dialdehyde
MJ	Methyl jasmonate
MS	Murashige and Skoog’s medium
PAL	Phenylalanine ammonia-lyase
PGR	Plant growth regulator
PINO	Pinoresinol
PLR	Pinoresinol reductase
PSS	Piperitol/sesamin synthase
PTOX	Podophyllotoxin
SA	Salicylic acid
SECO	Secoisolariciresinol
SNP	Single-nucleotide polymorphism
SOD	Superoxide dismutase
TAL	Tyrosine ammonia-lyase
TPC	Total phenolic content
UPLC	Ultra-high-performance liquid chromatography
WPM	Woody Plant Medium
YE	Yeast extract
